# Topical eye treatment with JGRi1, a protein/protein interaction inhibitor, mitigates retinal degeneration

**DOI:** 10.1038/s41419-026-08717-x

**Published:** 2026-04-15

**Authors:** Marco Cimino, Jack Serkiz, Joanne K. Konstantopoulos, Annamaria Tisi, Pamela Cappelletti, Rita Maccarrone, Rebecca M. Sappington, Marco Feligioni

**Affiliations:** 1 EBRI Rita Levi-Montalcini Foundation, Rome, Italy; 2 Translational Eye and Vision Research Center, Wake Forest University School of Medicine, Winston-Salem, NC USA; 3 Department of Biochemistry, Wake Forest University School of Medicine, Winston-Salem, NC USA; 4 Department of Science and Biomedical Technology, University of L’Aquila, L’Aquila, Italy; 5 Department of Neurorehabilitation Sciences, Casa Cura Igea, Milan, Italy

**Keywords:** Retina, Neurochemistry, Target validation, Recombinant peptide therapy

## Abstract

Retinal diseases (RDs) involve the degeneration of retinal cells, particularly retinal ganglion cells (RGCs), often driven by glutamate imbalance and aberrant signaling. We previously identified a presynaptic self-amplifying mechanism of glutamate overflow, where NMDA overstimulation activates JNK2-mediated phosphorylation of STX1A. To block this mechanism, a cell-permeable peptide, called JGRi1, was previously developed to disrupt the JNK2–STX1A interaction. Here, we investigated whether the inhibition of this pathway by JGRi1 could provide neuroprotection in retinal degeneration. We showed that JGRi1 efficiently reached the mouse retina upon topical administration as eye drops and granted retinal protection. Using an ex vivo optic nerve cut (evONC) model, we demonstrated that JGRi1 preserved RGC viability, reduced phosphorylation of JNK and STX1A, and lowered glutamate release. In retinal wholemounts, JGRi1 similarly preserved RGC survival. Furthermore, in an NMDA-induced degeneration model, JGRi1 protected RGCs, reduced glutamate levels, disrupted the JNK2–STX1A interaction, and limited microglial infiltration. Collectively, our findings highlight the central role of the JNK2–STX1A pathway in retinal degeneration and identify JGRi1 as a promising neuroprotective tool.

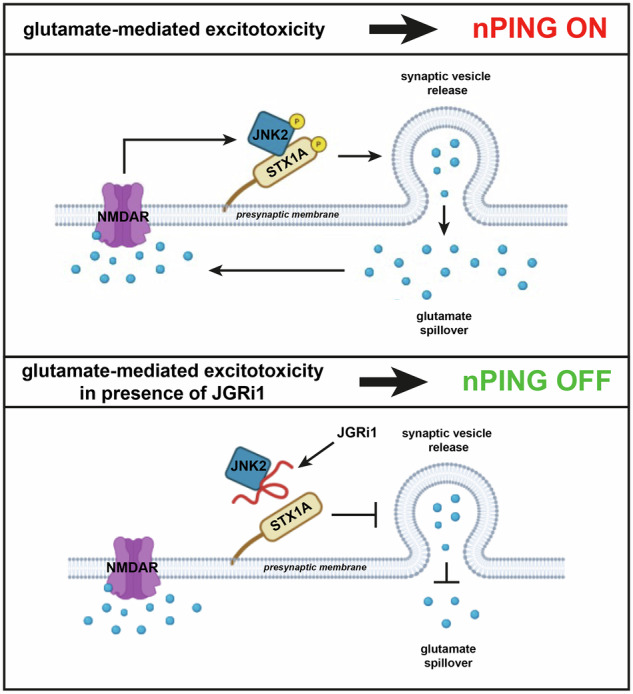

## Introduction

Glutamate excitotoxicity is a final common pathway in neurodegenerative diseases, yet clinical translation of neuroprotective strategies has consistently failed [1–3]. This is particularly true for retinal diseases (RDs), where the degeneration of retinal ganglion cells (RGCs) leads to irreversible vision loss [4, 5]. Dysregulation of glutamate causes the excessive activation of glutamate receptors, including N-methyl-D-aspartate (NMDA) receptors (NMDARs), with subsequent calcium overload, and neuronal injury [6]. Despite compelling evidence implicating glutamate imbalance in RDs across diverse animal models from glaucoma and diabetic retinopathy to retinal ischemia [7–10], and the neuroprotective effect shown by NMDAR antagonists in preclinical trials [11–14], the broad antagonism of NMDARs has proven clinically intractable. The failure of drugs like memantine underscores a fundamental limitation: suppressing essential synaptic NMDAR function causes unacceptable side effects, and systemic administration fails to achieve therapeutic retinal concentrations [15, 16]. However, targeting the NMDA cascade without interfering with the physiological function of NMDARs still remains a valid therapeutic strategy [16, 17].

A critical barrier has been the lack of selectivity in the inhibition between pre- and postsynaptic NMDARs by commercially available drugs. We previously identified a pathogenic presynaptic mechanism that may represent a more selective therapeutic target: the “non-canonical presynaptic-induced glutamate spillover” (nPING). In this self-amplifying circuit, glutamate overstimulation of presynaptic NMDARs triggers a JNK2-dependent phosphorylation of Syntaxin-1A (STX1A) [18–21]. This phosphorylation event, in turn, influences the binding of STX1A to other synaptic proteins, including Synaptosomal-Associated Protein, 25 kDa (SNAP25) and Synaptobrein-2 (VAMP2), which have both been shown to bind STX1A more efficiently when phosphorylated on Ser [14, 22, 23], and facilitates the mobilization of synaptic vesicles from reserve pools to active zones [24]. All these events enhance vesicular release, fueling further glutamate spillover and excitotoxic injury. This presynaptic positive-feedback loop represents a compelling target for intervention, as its disruption could quench excitotoxicity at its source while preserving physiological postsynaptic signaling.

To selectively dismantle this circuit, we developed JGRi1, a rationally designed cell-permeable peptide that acts as a protein-protein interaction (PPI) inhibitor to disrupt the JNK2-STX1A interaction. While our previous work established JGRi1’s efficacy in cellular and synaptosomal models [19, 20], its potential as a clinically viable neuroprotective agent remains unexplored. Critically, the translational success of any retinal therapy depends on effective drug delivery, with topical administration representing the ideal route for patient compliance and safety.

This work aimed at evaluating whether JGRi1, when topically administered as eye drops, can penetrate the eye and reach the retina and whether targeting the JNK2-STX1A interaction can grant neuroprotection in two distinct models of retinal degeneration, namely an ex vivo optic nerve cut model (evONC) and an intravitreal NMDA injection model, when administered topically in the form of eye drops.

## Results

### The ex vivo optic nerve cut model reduces RGC viability and increases L-glutamate immunoreactivity in the retina

**Fig. 1:**
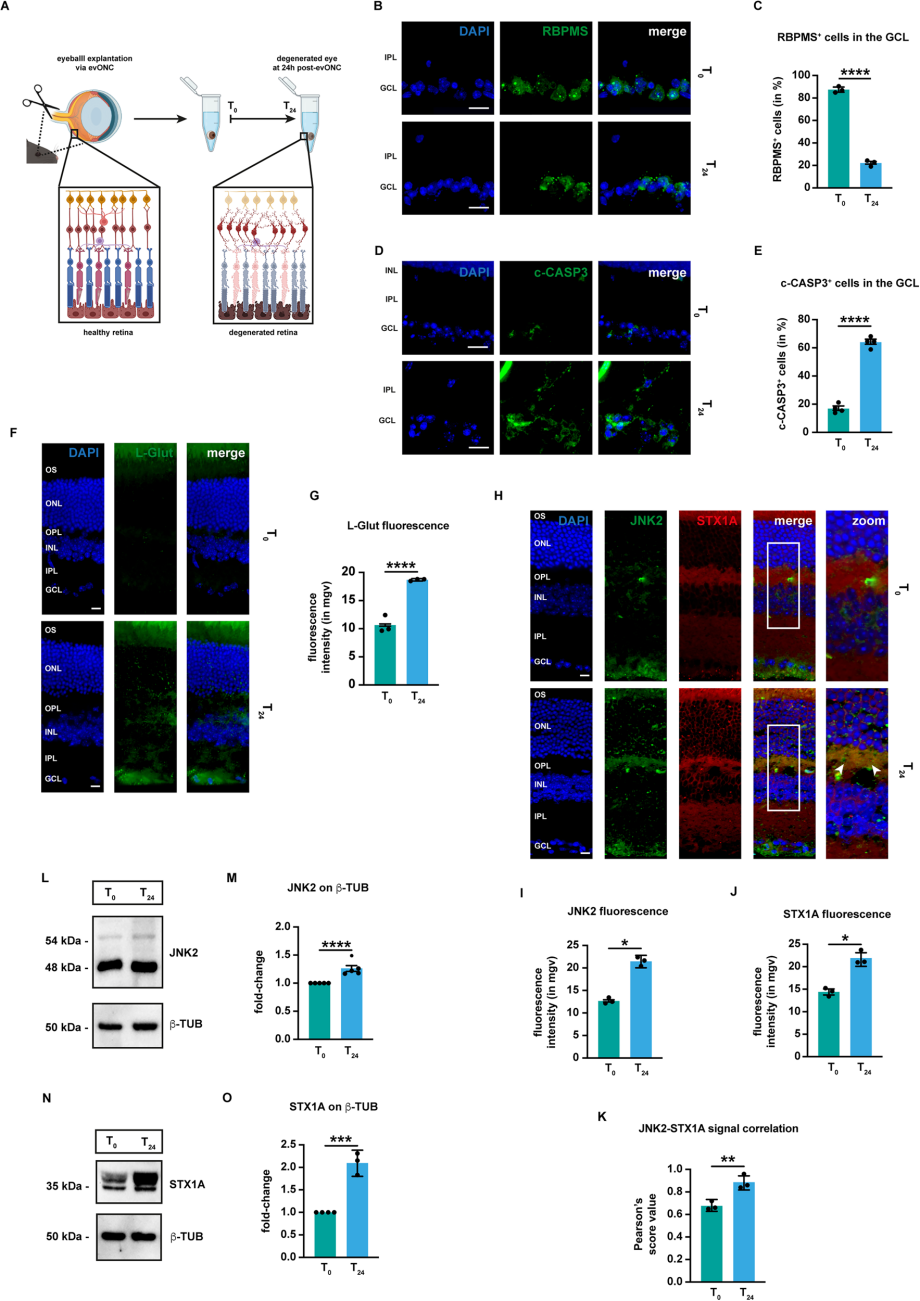
JNK2 and STX1A are induced and co-localize in retinas undergone evONC. **A** Schematic representation of the evONC model. **B** Representative immunofluorescence for RBPMS. Eyes were processed for immunofluorescence with an RBPMS-specific antibody (green). *n* = 4 independent experiments. **C** Quantification of RBPMS-positive cells. **D** Representative immunofluorescence for c-CASP3. Eyes were processed for immunofluorescence with a c-CASP3-specific antibody (green). *n* = 4 independent experiments. **E** Quantification of c-CASP3-positive cells. **F** Representative immunofluorescence for glutamate. Eyes were processed for immunofluorescence with a glutamate (L-glut) specific antibody (green). *n* = 4 independent experiments. **G** Quantification of the mean fluorescence intensity of L-glut. **H** Representative co-immunofluorescence for JNK2 and STX1A. Eyes were processed for immunofluorescence with a JNK2-specific antibody (green) and a STX1A-specific antibody (red). White arrowheads in the zoom column indicate areas where JNK2 and STX1A signals co-localize. *n* = 5 independent experiments. **I** Quantification of the mean fluorescence intensity of JNK2. **J** Quantification of the mean fluorescence intensity of STX1A. **K** Correlation analysis between JNK2 and STX1A signals. Pearson’s scores were calculated for each image from (**H**). **L** Representative western blot for JNK2. Samples were blotted, then incubated with primary antibodies against JNK2 and β-TUB (loading control). *n* = 4 independent experiments. **M** Densitometric analysis of JNK2 with respect to β-TUB. **N** Representative Western for STX1A. Samples were blotted, then incubated with primary antibodies against STX1A and β-TUB (loading control). *n* = 4 independent experiments. **O** Densitometric analysis of STX1A with respect to β-TUB. **Immunofluorescence caption:** OS outer segment, ONL outer nuclear layer, OPL outer plexiform layer, INL inner nuclear layer, IPL inner plexiform layer, GCL ganglion cell layer. 40X magnification. Scale bar 10 μΜ. **Bar plot caption:** Bar representing mean +/− S.D; number of individual replicates per condition shown in the scatter plot. Statistical analysis: one-tailed unpaired t-test, *p* < 0.05. *p* < 0.05. *p < 0.05; **p < 0.01; ***p < 0.001; ****p < 0.0001.

Previously, work from our group established a simple ex vivo model of retinal degeneration, as shown in Fig. 1A. Enucleated eyeballs, following a concomitant optic nerve cut (ONC), showed a powerful RGC loss associated with a strong reduction of their specific markers, such as Brain-Specific Homeobox/POU Domain Protein 3A (BRN3A) and Neuronal Nuclei Antigen (NeuN), upregulation of pro-apoptotic marker levels, including cleaved Caspase-3 (c-CASP3) (Fig. 1A) [25, 26].

Retinal tissue sections are frequently subjected to autofluorescence, due to the fact that the retina is rich in autofluorescent species, such as lipofuscin and melanin [27]. Therefore, to ascertain the reliability of our immunofluorescence data, immunofluorescence analysis was carried out on retinal slices without any antibody staining. Our analysis showed that retinal autofluorescence was mainly localized within the outer segment (OS) of photoreceptors (Fig. S1A). Therefore, the fluorescent signal in the OS will not be considered within this work. Additionally, to exclude possible artefacts deriving from secondary antibody cross-reactivity, retinal slices were incubated only with secondary antibodies. As shown in Fig. S1B, no antibody cross-reactivity was detectable within our samples.

In line with previous work, at 24 hours post-ONC (T_24_), we found that RGC viability was reduced in comparison to non-degenerated eyes (T_0_), as shown by the reduced immunoreactivity of the RGC marker RNA-Binding Protein with Multiple Splicing (RBPMS) in the ganglion cell layer (GCL) (Fig. 1B, C). Concomitantly, the c-CASP3-positive cells increased in the GCL (Fig. 1D, E).

Given the involvement of glutamate imbalance in the pathogenesis of several RDs [7–10], we investigated changes in glutamate (L-glut) immunoreactivity upon ONC. Our analysis showed that at T_0_ L-glut signal was poorly detectable, while at T_24_ L-glut appeared as a more punctate staining throughout the tissue, accumulating especially at the level of the GCL and the outer plexiform layer (OPL) (Fig. 1F). Quantification of the mean fluorescence intensity of L-glutamate validated our immunofluorescence findings (Fig. 1G).

### JNK2 and STX1A are upregulated in the ex vivo ONC mouse model

Afterwards, the expressions of both JNK2 and STX1A were evaluated in evONC retinas via co-immunofluorescence at both T_0_ and T_24_. As shown in Fig. 1H, the JNK2 signal was present mainly in the GCL at T_0_, although some signal was also detectable in the inner nuclear layer (INL) and outer plexiform layer (OPL); STX1A, on the other hand, was expressed throughout the retina, especially in the inner plexiform layer (IPL) and OPL. At T_24_, JNK2 signal strongly increased in the OPL, while STX1A immunoreactivity appeared higher in all retinal layers (Fig. 1I). In the OPL, STX1A exhibited a striking co-localization with JNK2 (Fig. 1H - merge, white arrowheads). The change in JNK2 and STX1A signals was assessed by measuring the mean fluorescence intensity, which confirmed the increase in the fluorescence intensity of both JNK2 (Fig. 1I) and STX1A (Fig. 1J). Ultimately, the co-localization between JNK2 and STX1A was assessed by calculating the Pearson’s score, showing an increase in the average Pearson’s score value at T_24_ (Fig. 1K).

Furthermore, the changes in expression of JNK2 and STX1A upon evONC were addressed by immunoblot. JNK2 at T_0_ appeared as two distinct bands, one with higher intensity with a molecular weight of around 46 kDa and the second one at around 54 kD, in line with previous reports [28]. At T_24_, there was an overall increase in the band signal intensity, which was statistically significant upon densitometric analysis (Fig. 1L, M). On the other hand, STX1A appeared as two very close bands at around 35 kDa. The intensity of these bands also increased upon evONC (Fig. 1N, O).

### NMDA treatment fosters RGC degeneration and glutamine synthetase expression in retinal wholemount preparations

The contribution of NMDA to retinal degeneration was assessed in cultured retinal wholemount preparations treated with NMDA (Fig. S2A, B). A 100 μM concentration of NMDA was selected based on previous studies performed on ex vivo explants treated with the same dose of NMDA [29, 30], whereas a 2-hour time point was selected to trigger glutamate release rather than chronic toxicity. Firstly, RGC viability was assessed by immunoblot for the RGC marker Brain-Specific Homeobox/POU Domain Protein 3A (BRN3A). At 24 hours post-mounting, there was a dramatic reduction of BRN3A expression, which was more pronounced upon treatment with 100 μM NMDA (Fig. S2C, D).

Additionally, the expression of glutamine synthetase (GS), one of the main enzymes involved in converting glutamate to glutamine [31], was assessed by immunoblot. The analysis revealed that at T_24_ there was an upregulation of GS expression, which was more marked by the addition of 100 μM NMDA (Fig. S2E, F).

### Intravitreal injection of NMDA induced retinal neurodegeneration

**Fig. 2:**
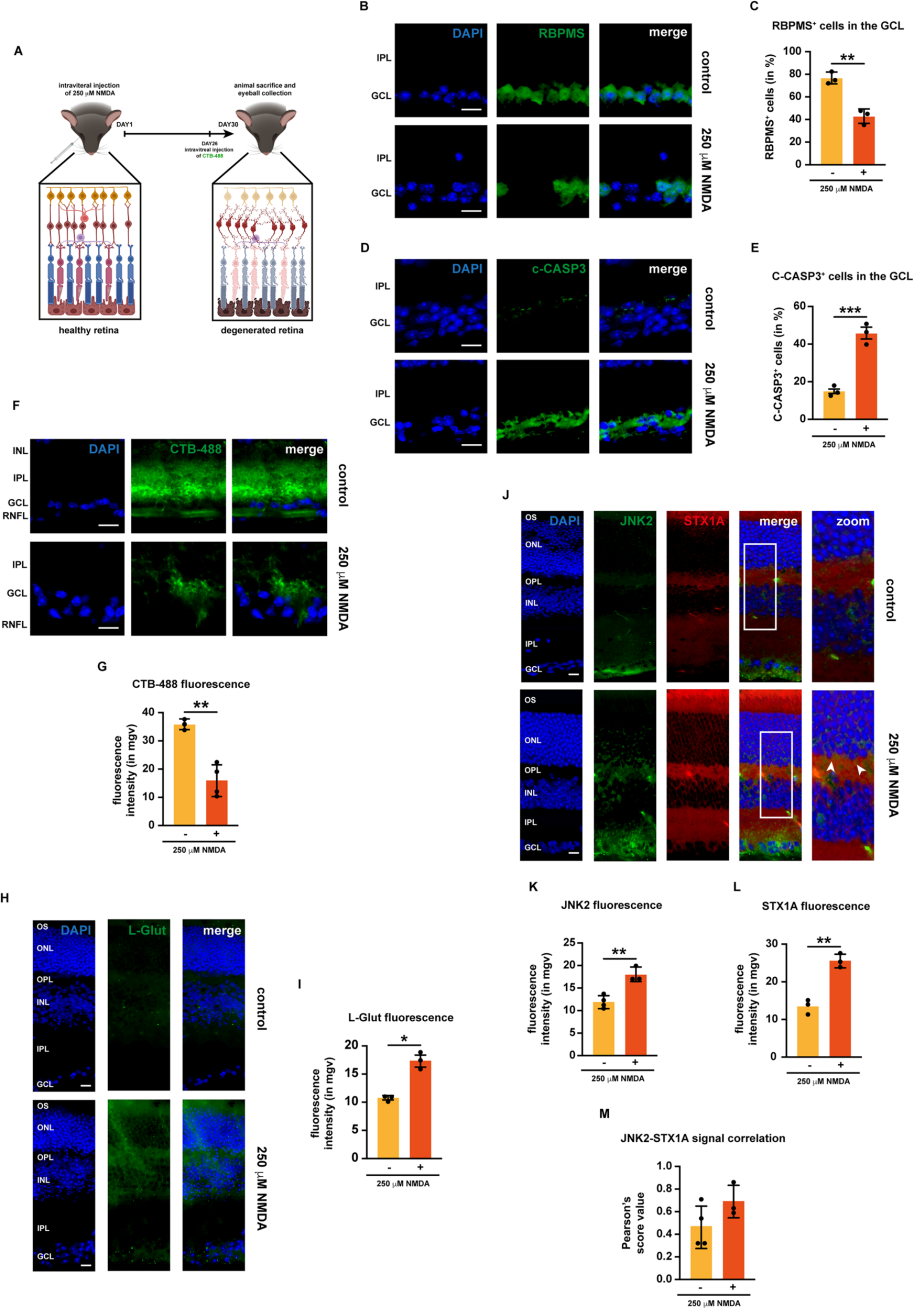
NMDA injection fosters retinal degeneration and induces JNK2 and STX1A expression. **A** Schematic representation of the NMDA injection model. **B** Representative immunofluorescence for NeuN. Eyes were processed for immunofluorescence with a NeuN-specific antibody (green). *n* = 4 independent experiments. **C** Quantification of NeuN-positive cells. **D** Representative immunofluorescence for c-CASP3. Eyes were processed for immunofluorescence with a c-CASP3-specific antibody (green). *n* = 4 independent experiments. **E** Quantification of c-CASP3-positive cells. **F** Representative immunofluorescence for CTB-488. **G** Quantification of the mean fluorescence intensity of CTB-488. **H** Representative immunofluorescence for glutamate. Eyes were processed for immunofluorescence with a glutamate (L-glut)-specific antibody (green). *n* = 4 independent experiments. **I** Quantification of the mean fluorescence intensity of L-glut. **J** Representative co-immunofluorescence for JNK2 and STX1A. Eyes were processed for immunofluorescence with a JNK2-specific antibody (green) and a STX1A specific antibody (red). White arrowheads in the zoom column indicate areas where JNK2 and STX1A signals strongly co-localize. *n* = 5 independent experiments. **K** Quantification of the mean fluorescence intensity of JNK2. **L** Quantification of the mean fluorescence intensity of STX1A. **M** Correlation analysis between JNK2 and STX1A signals. Pearson’s scores were calculated for each image from (**J**). **Immunofluorescence caption:** OS outer segment, ONL outer nuclear layer, OPL outer plexiform layer, INL inner nuclear layer, IPL inner plexiform layer, GCL ganglion cell layer. 40X magnification. Scale bar 10 μΜ. **Bar plot caption:** Bar representing mean +/− S.D; number of individual replicates per condition shown in the scatter plot. Statistical analysis: one-tailed unpaired t-test, *p* < 0.05. *p* < 0.05. *p < 0.05; **p < 0.01; ***p < 0.001.

Another well-established model for retinal degeneration is the NMDA-induced degeneration model which is known to promote RGC degeneration through NMDAR overactivation and glutamate overflow [7] (Fig. 2A). To test retinal neurodegeneration in this model both RGC viability and apoptosis were assessed by immunofluorescence. Indeed,30 days after NMDA injection, there was a reduction in RCG viability in comparison to untreated mice, as shown by both immunofluorescence for the RBPMS and confirmed by the quantification of RBPMS-positive cells in the GCL (Fig. 2B, C); conversely, the number of c-CASP3-positive cells in the GCL increased (Fig. 2D, E). To address the effect of NMDA injection on neuronal connections within the retina, 4 days prior to their sacrifice, mice were injected with fluorescently labeled cholera toxin B (CTB-488), which enabled anterograde tracing of RGC axons in the retinal nerve fiber layer (RFNL). In untreated retinas, CTB-488 was uptaken by the RGCs and effectively transported through their axons in the RFNL, whilst in NMDA-injected mice CTB-488 uptake and transport failed in RGCs, indicating RGC compromise following NMDA exposure (Fig. 2F) [32], which was also confirmed by the subsequent quantification of CTB-488 fluorescence (Fig. 2G).

To assess the effect of NMDA on retinal glutamate levels, immunofluorescence for L-glut was carried out. In untreated retinas, L-glutamate was poorly detectable, although some signal was detectable between the INL and the OPL; on the other hand, in NMDA-injected mice, an increase in L-glut immunoreactivity was observed both in the inner retina and the GCL (Fig. 2H, I).

### Intravitreal injection of NMDA promotes the expression of JNK2 and STX1A in the retina

The expression of JNK2 and STX1A was assessed in the retina upon NMDA-injection. In untreated mice, JNK2 expression was detectable mainly in the GCL, while, after the injection, JNK2 was detectable also in the IPL and OPL, with a few JNK2 signals appearing also in the INL and ONL (Fig. 2J). Quantification of the mean fluorescence intensity of JNK2 in the retina confirmed our initial finding (Fig. 2K). Similarly, STX1A immunoreactivity was also higher in the retinas of NMDA-injected mice. Like for JNK2, quantification of STX1A fluorescence in the retina confirmed the NMDA-induced increase of STX1A immunoreactivity (Fig. 2L). Afterwards, the JNK2-STX1A co-localization in the retina of NMDA-injected mice was analyzed via Pearson’s analysis, which showed that upon NMDA injection, there was a slight, yet no significant, increase in the co-localization between the two proteins (Fig. 2M).

### JGRi1 effectively reaches the retina upon ex vivo and in vivo administration

In previous works, we have shown that the cell-permeable peptide JGRi1 prevented the JNK2-STX1A interaction, as well as glutamate spillover in multiple models [19, 20]. On these premises, we decided to test the potential neuroprotective effect of JGRi1 in our experiments.

Firstly, the eye permeability of JGRi1 was evaluated upon ex vivo treatment. Enucleated mouse eyeballs were immersed in balanced salt solution (BSS) buffer and incubated with different dosages (50 μM, 250 μM, 500 μM) of fluorescein (FITC)-tagged JGRi1 (F-JGRi1) for 1 hour at 37 °C (Fig. S3A). As a control, we included a version of the peptide devoid of the HIV-Tat sequence (ΔTat-F-JGRi1), with consequent lower cell permeability. Immunofluorescence analysis revealed that F-JGRi1 was able to reach the retina upon topical application. F-JGRi1 was detected in multiple layers, including GCL, the IPL, and the OPL (Fig. S3B). Conversely, incubation of enucleated eyeballs with ΔTat-F-JGRi1 resulted in poor accumulation of F-JGRi1 in the retina (Fig. S3B). Quantification of the F-JGRi1 signal showed that the peptide accumulated in both the OPL and the GCL in a dose-dependent manner, whereas ΔTat-F-JGRi1 did not (Fig. S3C, D). Then, the number of FITC-positive cells in the GCL was counted (Fig. S3E). The analysis showed a dose-dependent increase of FITC-positive cells in the GCL. Conversely, ΔTat-F-JGRi1 treatment led to no accumulation of the peptide (Fig. S3F).

**Fig. 3:**
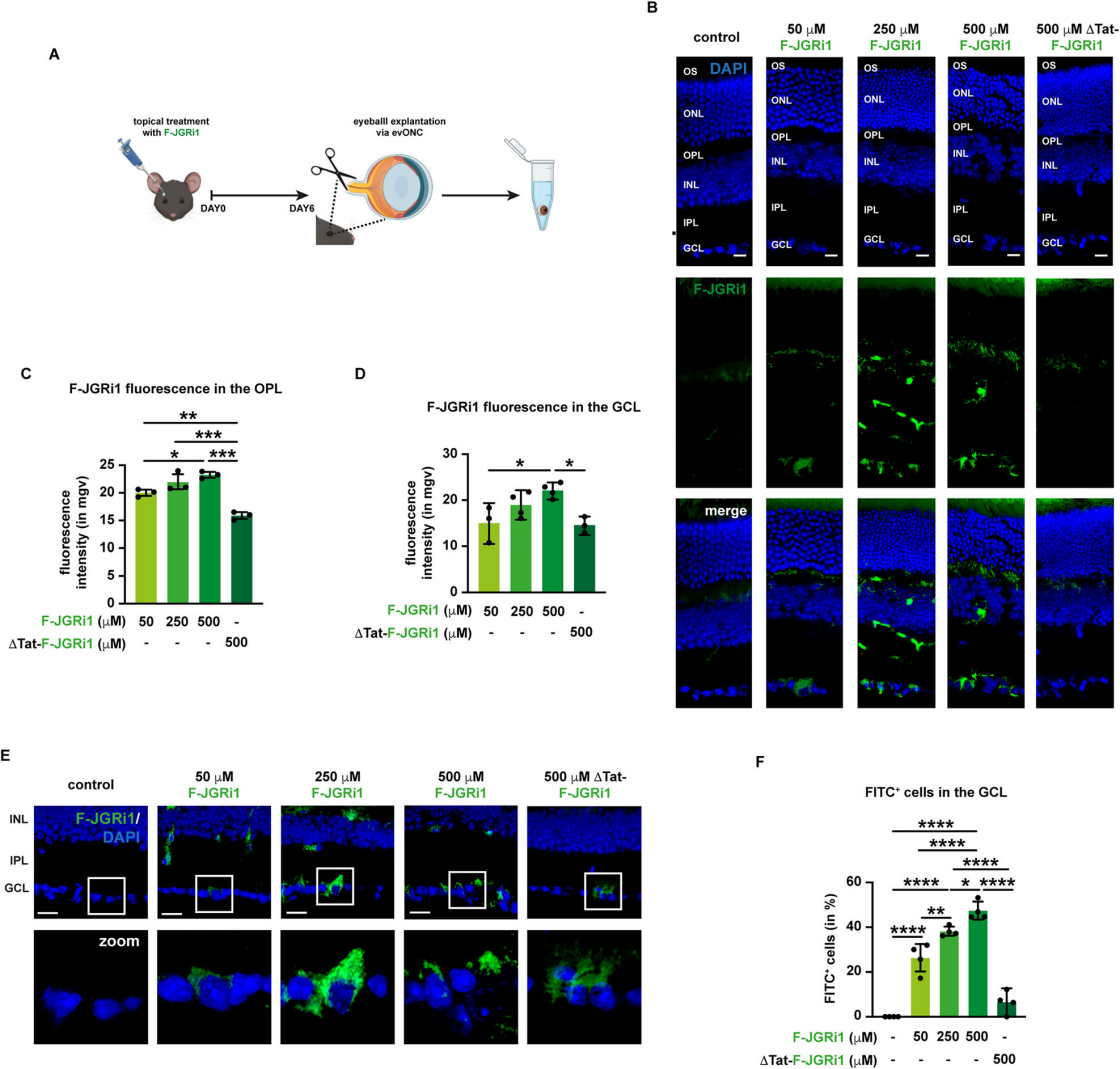
F-JGRi1 penetrates the eye and accumulates in the retina of C57BL/6 J mice upon topical administration for 6 days. **A** Schematic representation of in vivo F-JGRi1 treatment. **B** Representative immunofluorescence for F-JGRi1. *n* = 5 independent experiments. **C** Quantification of the mean fluorescence intensity of F-JGRi1 in the OPL. **D** Quantification of the mean fluorescence intensity of F-JGRi1 in the GCL. **E** Enlarged view of F-JGRi1 accumulation in the GCL of treated mice. *n* = 5 independent experiments. **F** Quantification of F-JGRi1-positive cells. **Immunofluorescence caption:** OS outer segment, ONL outer nuclear layer, OPL outer plexiform layer, INL inner nuclear layer, IPL inner plexiform layer; GCL, ganglion cell layer. 40X magnification. Scale bar 10 μΜ.**Bar plot caption:** Bar representing mean +/− S.D; number of individual replicates per condition shown in the scatter plot. Statistical analysis: One-way ANOVA, two-tailed *post hoc* Tukey test, *p* < 0.05. *p* < 0.05. *p < 0.05; **p < 0.01; ***p < 0.001; ****p < 0.0001.

Afterwards, the eye permeability of JGRi1 was also tested upon in vivo treatment as eye-drops. For this purpose, C57BL/6 J mice were treated with different dosages of F-JGRi1 (50 μM, 250 μM, 500 μM) dissolved into BSS buffer for 6 days, one drop per die. ΔTat-F-JGRi1 was included as a control (Fig. 3A). Similarly to the ex vivo treatment experiment, topically administered F-JGRi1 successfully accumulated in the retina, especially in the GCL and in the OPL (Fig. 3B). Of note, differently from the ex vivo treatment, some ΔTat-F-JGRi1 accumulated only in the GCL upon treatment (Fig. 3B). Quantification of the F-JGRi1 accumulation in the retina showed a dose-dependent trend both as raw fluorescence of the peptide (Fig. 3C, D), as well as FITC-positive cells in the GCL (Fig. 3E, F).

### Topical JGRi1 treatment does not affect normal JNK2 and STX1A expression as well as retinal glutamate levels

To study the effect of JGRi1 on JNK2 and STX1A physiology, in the absence of any retinal damage, C57BL/6 J mice were treated for 6 days with different dosages of JGRi1 (50 μM, 250 μM, 500 μM), one drop per day (Fig. S4A). A version of JGRi1 in which the effector sequence, but not the Tat, was completely scrambled up (sJGRi1) was included as an additional control during the treatment.

Of note, at the end of the treatment, after animal sacrifice, eye bulbs were observed under a light microscope for retinal collection. No apparent opacity of cornea or lens was observed in all conditions tested. Afterwards, eyes underwent co-immunofluorescence analysis for both JNK2 and STX1A. The analysis showed that the JNK2 signal was present mainly in the GCL, while STX1A was highly expressed in the plexiform layers of the retina (Fig. S4B). Interestingly, treatment with increasing dosages of JGRi1 did not produce any effect on either JNK2 or STX1A expression, as the two proteins retained their normal tissue distribution and no changes were observed in the intensity of their fluorescent signals upon treatment (Fig. S4C, D). Additionally, the different JGRi1 doses did not affect co-localization of the two proteins, as shown by the Pearson’s score calculation (Fig. S4E).

Next, the effect of JGRi1 on physiological glutamate levels was assessed via immunofluorescence analysis using the L-glut specific antibody, which showed no apparent differences triggered by increasing dosages of JGRi1 (Fig. S4F). Such observation was later confirmed by quantification of retinal L-glut fluorescence upon treatment (Fig. S4G).

### Topical treatment with JGRi1 protects RGC from evONC-induced degeneration

**Fig. 4:**
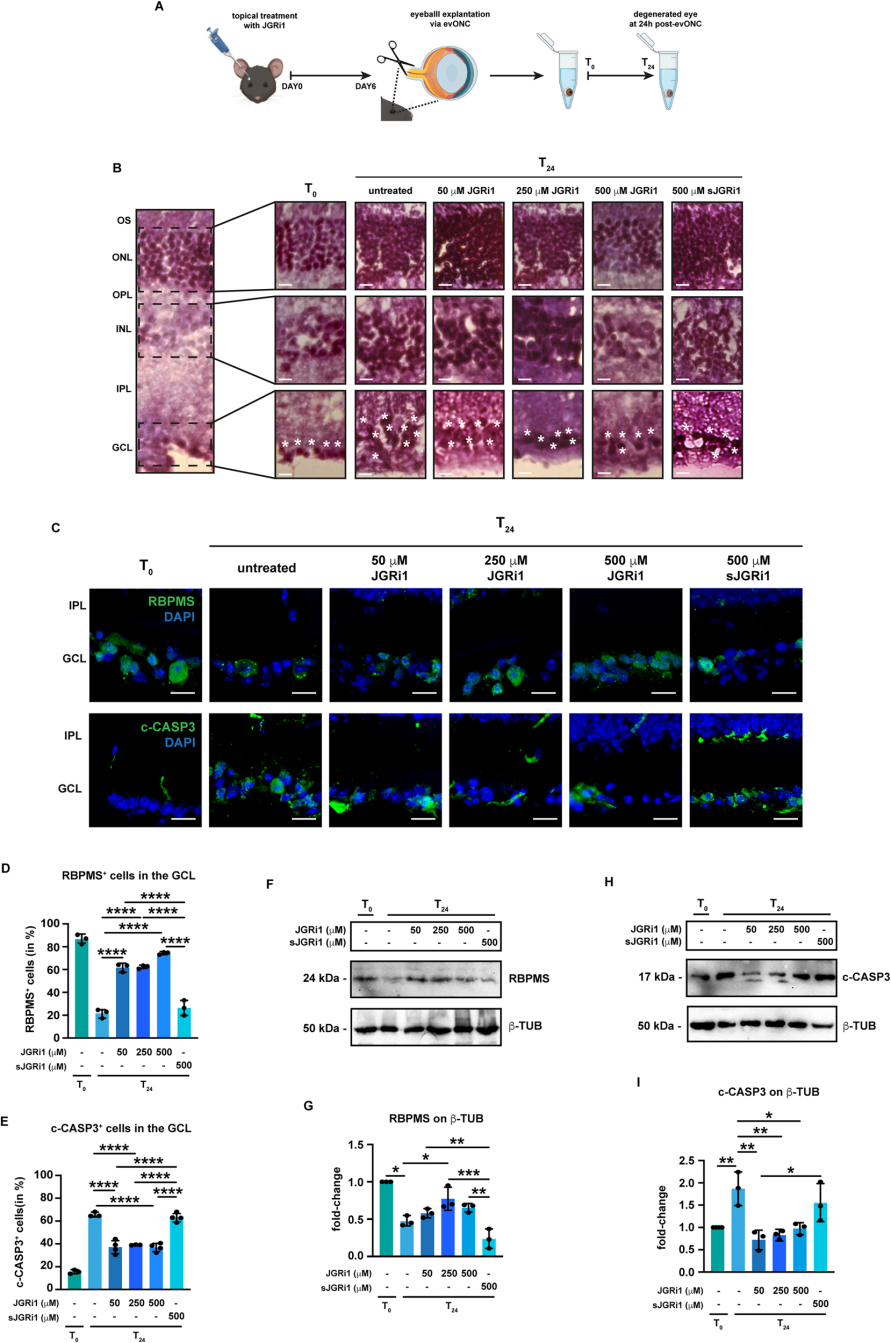
JGRi1 protects retinas and RGCs from evONC-induced neurodegeneration. **A** Schematic representation of JGRi1 treatment. **B** Representative hematoxylin-eosin staining. Eyes were stained with hematoxylin, counterstained with eosin. A sample retina is shown in the left part of the panel. For each nuclear layer, an enlarged view is provided. White asterisks indicate RGCs. *n* = 3 independent experiments. **C** Representative immunofluorescence for (upper row) and c-CASP3 (bottom row) on retinal slices from the evONC model. Eyes were processed for immunofluorescence with a specific antibody (green). *n* = 4 independent experiments. **D** Quantification of RBPMS-positive cells. For the reader’s convenience, statistical significance only in comparison to T_24_ and between active JGRi1 and sJGRi1 is shown. **E** Quantification of c-CASP3-positive cells. For the reader’s convenience, statistical significance only in comparison to T_24_ and between active JGRi1 and sJGRi1 is shown. **F** Representative western blot for RBPMS. Samples were blotted, then incubated with primary antibodies against RBPMS and β-TUB (loading control). *n* = 4 independent experiments. **G** Densitometric analysis of RBPMS with respect to β-TUB. **H** Representative Western blot showing the expression of c-CASP3 in retinas from the evONC model. Samples were blotted, then incubated with primary antibodies against c-CASP3 and β-TUB (loading control). *n* = 5 independent experiments. **I** Densitometric analysis of c-CASP3 with respect to β-TUB. **Immunofluorescence caption:** OS outer segment, ONL outer nuclear layer, OPL outer plexiform layer, INL inner nuclear layer, IPL inner plexiform layer; GCL, ganglion cell layer. 40X magnification. Scale bar 10 μΜ. **Bar plot caption:** Bar representing mean +/− S.D; number of individual replicates per condition shown in the scatter plot. Statistical analysis: One-way ANOVA, two-tailed *post hoc* Tukey test, *p* < 0.05. *p* < 0.05. *p < 0.05; **p < 0.01; ***p < 0.001; ****p < 0.0001.

Next, the protective effect of JGRi1 was assessed in the evONC model. C57BL/6J mice were treated with one drop daily for 6 days with JGRi1 at different concentrations (50 μM, 250 μM, and 500 μM). Afterwards, animals were sacrificed, and eye bulbs were processed for evONC. The sJGRi1 was included as a control (Fig. 4A).

Firstly, hematoxylin/Eosin staining was carried out to evaluate the retinal cytoarchitecture. At T_24,_ there was a loss of integrity within all three nuclear layers of the retina, which was progressively rescued by treatment with increasing dosages of JGRi1 (Fig. 4B).

Immunofluorescence for RBPMS was carried out to evaluate RGC viability. As already shown in Fig. 1B, C, at T_24,_ there was a loss of RGCs in comparison to T_0_. The treatment with all three doses of JGRi1 caused an increase in the number of RBPMS-positive cells in the GCL, with no significant differences between all the tested doses. As expected, 500 μM sJGRi1 failed to rescue RGC viability upon evONC (Fig. 4C, D). RBPMS expression was also evaluated by immunoblot. RBPMS appeared as a single band at around 24 kDa of molecular weight. Densitometric analyses showed that RBPMS was reduced at T_24_, while JGRi1 led to a recovery of the RBPMS signal, although only 250 μM JGRi1 provided statistical significance (Fig. 4F, G). Treatment with sJGRi1 did not produce any protective effect on RGC viability in comparison to the active peptide. Similarly, the number of apoptotic cells in the GCL was addressed by immunofluorescence for c-CASP3. At T_24_, the number of c-CASP3-positive cells increased, but treatment with all dosages of JGRi1 reduced it in a significant manner (Fig. 4C–E). In parallel, c-CASP3 expression was evaluated by immunoblotting. Immunoblot for c-CASP3 yielded a single band with a molecular weight of 17 kDa. c-CASP3 expression increased at T_24_, while it was reduced significantly by all three doses of JGRi1, but not sJGRi1, in line with the immunofluorescence reports. (Fig. 4H, I).

### Topical JGRi1 reduces the evONC-induced glutamate release in the retina

**Fig. 5:**
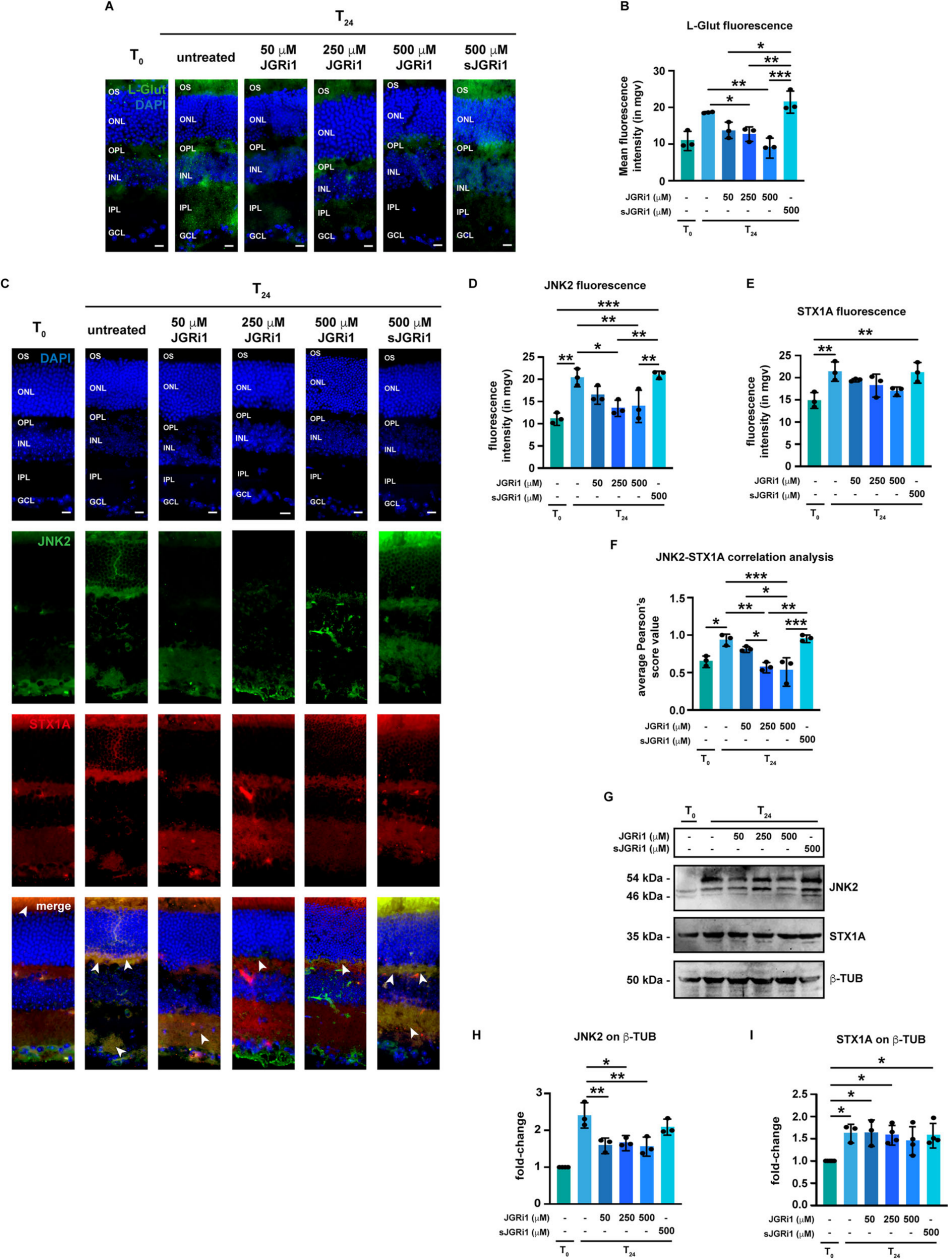
Effect of JGRi1 on retinal glutamate levels and the expression JNK2 and STX1A in evONC model. **A** Representative immunofluorescence for L-glut. Eyes were processed for immunofluorescence with an L-glut-specific antibody (green). *n* = 3 independent experiments. **B** Quantification of the mean fluorescence intensity of L-glut. **C** Representative co-immunofluorescence for JNK2 and STX1A on retinal slices from evONC model. Eyes were processed for immunofluorescence with a JNK2 specific antibody (green) and a STX1A specific antibody (red). White arrowheads in the merge column indicate areas where JNK2 and STX1A signals co-localize. *n* = 5 independent experiments. **D** Quantification of the mean fluorescence intensity of JNK2. **E** Quantification of the mean fluorescence intensity of STX1A. **F** Correlation analysis between JNK2 and STX1A signals in retinal slices from evONC. Pearson’s scores were calculated for each image from (**C**). **G** Representative western blot showing the expression of JNK2 and STX1A in retinas from the evONC model. Samples were blotted, then incubated with primary antibodies against JNK2, STX1A, and β-TUB (loading control). *n* = 5 independent experiments. **H** Densitometric analysis of JNK2 with respect to β-TUB. For the reader’s convenience, statistical significance only in comparison to T_24_ and between active JGRi1 and sJGRi1 is shown. **I** Densitometric analysis of STX1A with respect to β-TUB. **Immunofluorescence caption:** OS outer segment, ONL outer nuclear layer, OPL outer plexiform layer, INL inner nuclear layer, IPL inner plexiform layer, GCL ganglion cell layer. 40X magnification. Scale bar 10 μΜ. **Bar plot caption:** Bar representing mean +/− S.D; number of individual replicates per condition shown in the scatter plot. Statistical analysis: One-way ANOVA, two-tailed *post hoc* Tukey test, *p* < 0.05. *p* < 0.05. *p < 0.05; **p < 0.01; ***p < 0.001.

The effect of JGRi1 on the evONC-induced release of glutamate was addressed by immunofluorescence. At T_24_, as expected, L-glut immunoreactivity was higher throughout the retina (Fig. 5A), but treatment with JGRi1 reduced it. Of note, only 250 μM and 500 μM JGRi1 produced a significant reduction in retinal glutamate immunoreactivity, with the latter bringing it even below control levels (Fig. 5B).

### Topical JGRi1 protects the INL from degeneration upon evONC

In the retinal circuitry, the INL accounts for the bridge between photoreceptors and GCL. The INL hosts multiple cell types, including amacrine, horizontal, and bipolar cells. In particular, a specific subtype of bipolar cells, namely rod bipolar cells, which sum signals from many rods and convey them to the GCL [33], and A2 amacrine cells, which are key interneurons for rod-mediated vision [34], have been shown to be selectively vulnerable to glaucomatous degeneration [34, 35]. Therefore, the effect of JGRi1 on the viability of these cell types was assessed by immunofluorescence using the A2 amacrine cell and rod bipolar cell marker Prospero homeobox protein 1 (PROX1) [36–38]. The immunofluorescence highlighted the presence of PROX1-positive cells in the INL, which hosts this retinal subpopulation (Fig. S5A). At T_24_, the number of PROX1-positive cells in the INL was almost halved (Fig. S5A, B); however, treatment with 500 μM JGRi1, but not 250 μM or 50 μM JGRi1, was able to rescue cell number, although to a small extent. As expected, sJGRi1 did not produce any protective effect on PROX1-positive cell number (Fig. S5A, B).

### Topical JGRi1 reduces the evONC-induced glutamate accumulation in the synaptic layers of the retina

Rod bipolar cells receive glutamatergic projections from rods and, in turn, engage glutamatergic connections with the GCL. These connections are fundamental for night vision, which is severely compromised in early-stage of glaucoma [39, 40], probably due to the glutamate-mediated degeneration of cells involved in this cascade. Thus, to evaluate the potential accumulation of glutamate at the level of synapses, especially those between bipolar cells and RGCs, and to measure the effect of JGRi1 on it, a co-immunostaining for both L-glut and the presynaptic marker synaptotagmin-1 (SYT1) was performed [41]. SYT1 immunoreactivity was particularly abundant in the OPL and the IPL and overlapped with the L-glut signal in these layers (Fig. S5C). The overlap between the two signals was confirmed by the subsequent Pearson’s analysis (Fig. S5D). The co-localization was higher at T_24_, especially in the IPL, where the synapses between bipolar cells and RCG lie, suggesting that glutamate was actually accumulating at the synaptic level; interestingly, treatment with JGRi1, but not sJGRi1, progressively reduced L-glut immunoreactivity, as well as its co-localization with SYT1, although only 500 μM JGRi1 produced a significant effect (Fig. S5C, D).

### Topical administration of JGRi1 reduces the upregulation of JNK2 but not STX1A, as well as their retinal co-localization induced by evONC

To evaluate the effect of JGRi1 on JNK2 and STX1A expression in the retina, evONC eyes were first treated with the aforementioned doses of JGRi1 and sJGRi1, then processed for co-immunofluorescence using JNK2- and STX1A-specific antibodies. As shown previously, at T_24,_ JNK2 immunoreactivity in the inner retina increased. Treatment with JGRi1 led to a decrease of JNK2 immunoreactivity throughout the tissue: indeed, while 50 μM JGRi1 only produced a mild reduction in JNK2 immunoreactivity, 250 μM JGRi1 restored a pattern of JNK2 expression very close to that seen at T_0_, although some JNK2 signal remained detectable in the inner retina. Ultimately, treatment with 500 μM JGRi1 reduced JNK2 immunoreactivity in the GCL, although the dome signal was still detectable between the OPL and the INL (Fig. 5C). Likewise, STX1A expression increased at T_24_, but, unlike JNK2, no significant reduction in STX1A level was found in response to all the tested dosages of JGRi1 (Fig. 5C). Such observations were also validated by measuring the fluorescence intensity of both proteins (Fig. 5D, E). As expected, pre-treatment with sJGRi1 does not have any effect on JNK2 nor STX1A expression at T_24_.

As shown in Fig. 1H, JNK2 and STX1A signals strongly co-localized in the OPL at T_24_. To address the effect of JGRi1 on the evONC-fostered co-localization between JNK2 and STX1A, co-immunofluorescence for JNK2 and STX1A was carried out. The analysis showed that JGRi1 led to a dose-dependent reduction of the JNK2-STX1A co-localization. In fact, while with 50 μM JGRi1, JNK2 and STX1A still co-localized in the IPL, treatment with 250 μM and 500 μM JGRi1 resulted in a minimal overlap between their signals (Fig. 5C – white arrowheads). Interestingly sJGRi1 treatment produced an even more marked co-localization between JNK2 and STX1A (Fig. 5C). Such observations were validated by running the Pearson’s analysis for both proteins (Fig. 5F).

Afterwards, protein levels of JNK2 and STX1A were both assessed by immunoblot. JNK2 expression, which was higher at T_24_, was reduced by all dosages of JGRi1, but not by sJGRi1 (Fig. 5G). However, the expression of JNK2 was still higher than that of T_0_, probably as a result of the fact that the 54 kDa band was still detectable in any condition (Fig. 5G, H). Similarly to JNK2, immunoblot for STX1A also confirmed our immunofluorescence reports, with none of the tested JGRi1 doses able to bring back STX1A expression to control levels (Fig .5G–-I).

### Topical administration of JGRi1 reduces the phosphorylation of both JNK2 and STX1A and the rate of SNARE complex formation induced by evONC

**Fig. 6:**
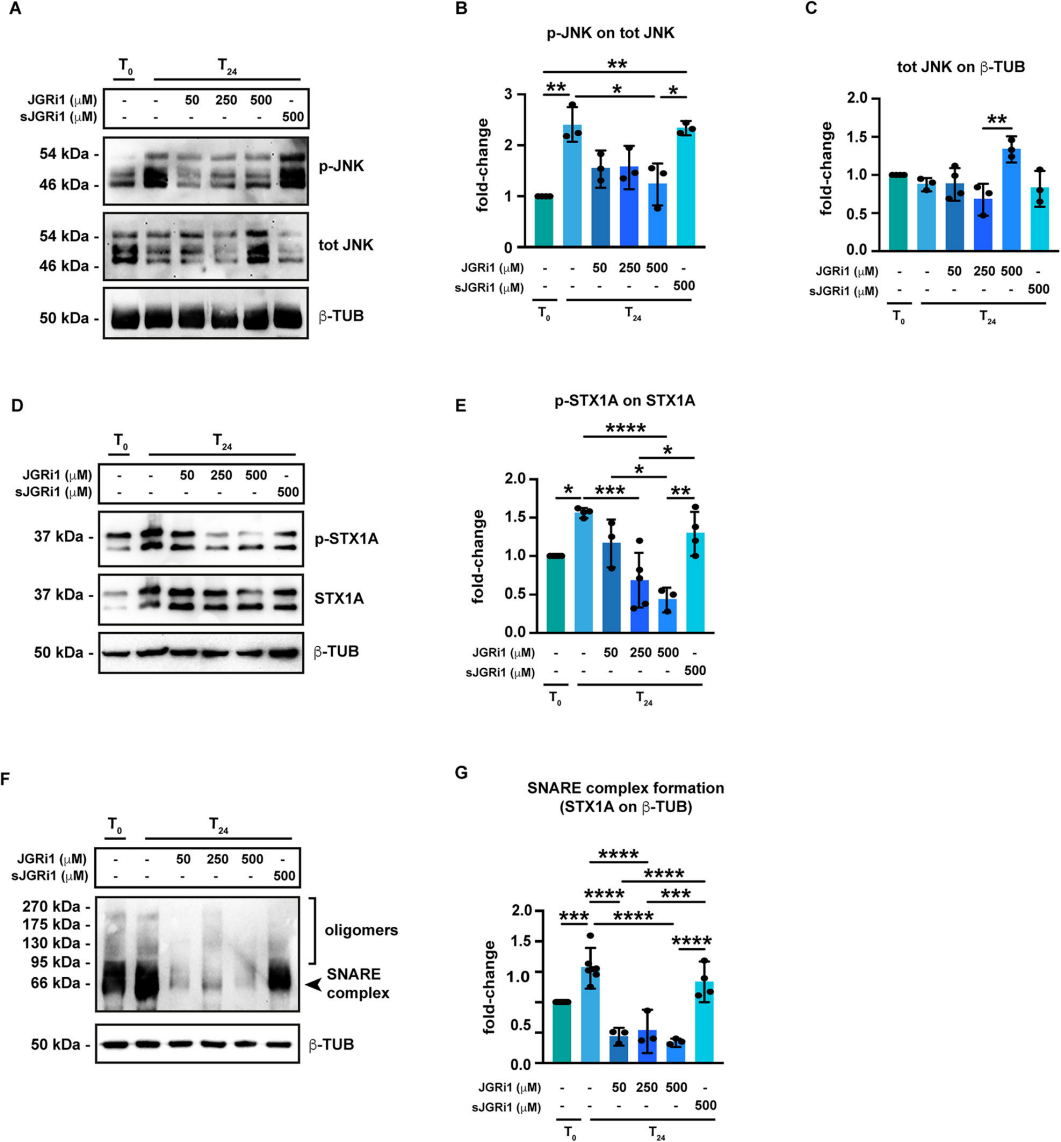
Effect of JGRi1 on the phosphorylation of JNK2 and STX1A and on SNARE complex formation in evONC model. **A** Representative western blot showing the p-JNK. Samples were blotted, then incubated with primary antibodies against p-JNK, total JNK, and, ultimately, β-TUB (loading control). n = 5 independent experiments. **B** Densitometric analysis of p-JNK with respect to total JNK. **C** Densitometric analysis of total JNK with respect to β-TUB. **D** Representative western blot showing the p-STX1A. Samples were blotted, then incubated with a specific primary antibody against p-STX1A, then STX1A and, ultimately, β-TUB (loading control). *n* = 5 independent experiments. **E** Densitometric analysis of p-STX1A with respect to STX1A. **F** Representative western blot showing SNARE complex formation in retinas from the evONC model. Non-boiled protein samples were blotted, then incubated with primary antibodies against SNARE complex component STX1A and β-TUB (loading control). *n* = 6 independent experiments. **G** Densitometric analysis of STX1A with respect to β-TUB. For the reader’s convenience, statistical significance only in comparison to T_24_ and between active JGRi1 and sJGRi1 is shown. **Bar plot caption:** Bar representing mean +/− S.D; number of individual replicates per condition shown in the scatter plot. Statistical analysis: One-way ANOVA, two-tailed *post hoc* Tukey test, *p* < 0.05. *p* < 0.05. *p < 0.05; **p < 0.01; ***p < 0.001; ****p < 0.0001.

In our previous works, we reported that in the evONC mouse model, the phosphorylation of JNK (p-JNK) was increased within the retina [25, 26]. Therefore, the effect of JGRi1 treatment on the evONC-induced JNK phosphorylation was assessed by immunoblot. At T_24,_ the phosphorylation of JNK was strongly induced; the treatments with all doses of JGRi1, but not sJGRi1, reduced the JNK phosphorylation with significance reached only upon 500 μM JGRi1 treatment (Fig. 6A, B). Notably, treatment with 500 μM JGRi1 induced a significant upregulation of total JNK levels in the immunoblot (Fig. 6A–C). Afterwards, STX1A phosphorylation at Ser 14 was also investigated. As said previously, such residue is specifically phosphorylated by JNK2 during excitotoxic stress, thus favoring the release of glutamate [19, 20]. In the immunoblots, phosphorylated STX1A (p-STX1A) appeared as two distinct bands of around 35 kDa (Fig. 6D); at T_24_ p-STX1A signal was higher than T_0_, as confirmed by densitometric analysis; p-STX1A levels were lowered only by treatments with 250 μM JGRi1 and 500 JGRi1 (Fig. 6E).

The release of glutamate entails the formation of Soluble N-ethylmaleimide-Sensitive Factor Attachment Proteins Receptor (SNARE) complexes, which are multiprotein complexes that drive the fusion of neurotransmitter-storing vesicles to the plasma membrane [42, 43]. Therefore, the rate of SNARE complex formation in the retina was assessed in the evONC model. Incubation with STX1A antibody revealed the presence of a band at around 66 kDa, corresponding to the core SNARE complex, plus a high molecular weight protein smear, corresponding to its oligomers (Fig. 6F), as previously reported [44]. The intensity of the signal increased at T_24,_ indicating that evONC boosted SNARE complex formation, while all the doses of JGRi1 dramatically reduced it. The treatment with sJGRi1 did not produce any effect on SNARE complex formation (Fig. 6G).

### JGRi1 treatment reduced NMDA-induced RGC death in retinal wholemount preparations

To study the protective effect of JGRi1 on retina wholemounts, preparations were treated with 1 μM JGRi1 for 1 hour prior to NMDA stimulation. (Fig. S6A). As shown previously, at T_24_ BRN3A reduction was enhanced by treatment with 100 μM NMDA. However, treatment with 1 μM JGRi1 prior to NMDA was able to rescue the NMDA-enhanced reduction of BRN3A expression, bringing it back almost to control levels (Fig. S6B, C). Moreover, our previous data showed that at T_24,_ 100 μM NMDA treatment produced an increase in GS expression. Treatment with 1 μM JGRi1 reduced GS levels in comparison only to NMDA-treated retinas, but not retinas at T_24_ (Fig. S6B, D).

### Topical JGRi1 protects the GCL and reduces glutamate release in the mouse NMDA-induced retinal degeneration model

**Fig. 7:**
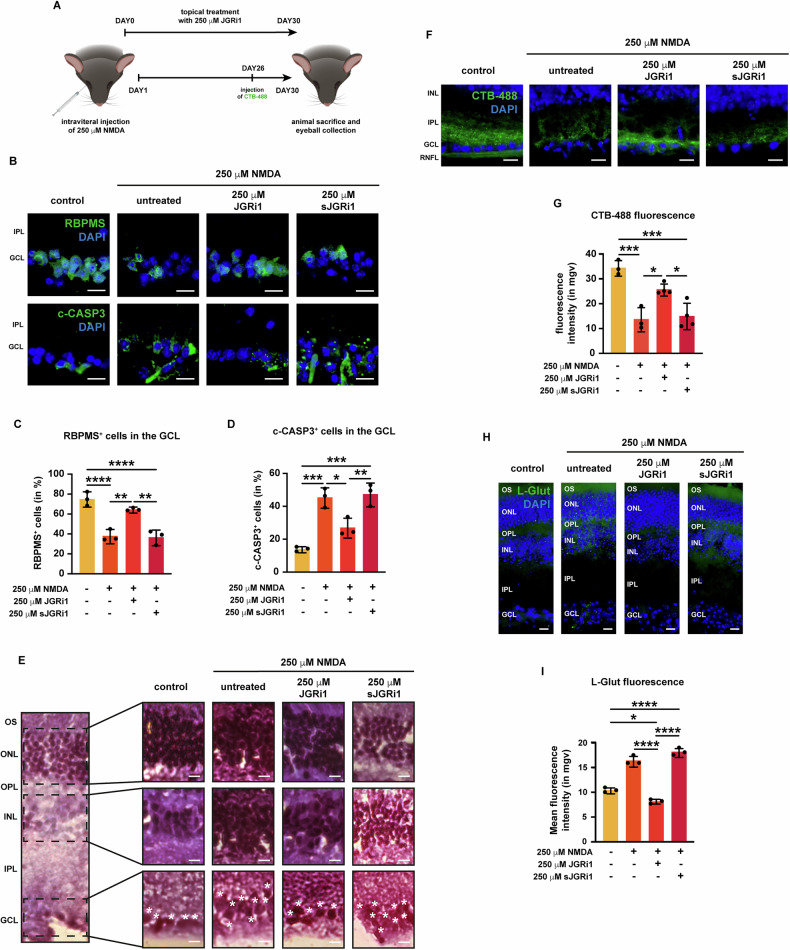
JGRi1 protects retinas from NMDA-induced degeneration. **A** Schematic representation of JGRi1 treatment. **B** Representative immunofluorescence for NeuN (upper row) and c-CASP3 (bottom row). Eyes were processed for immunofluorescence with specific antibody (green). *n* = 4 independent experiments. **C** Quantification of NeuN-positive cells. **D** Quantification of c-CASP3-positive cells. **E** Representative hematoxylin-eosin staining on retinal slices from NMDA-injected mice. Eyes were stained with hematoxylin, then counterstained with eosin. A sample retina is shown in the left part of the panel. For each nuclear layer an enlarged view is provided. White asterisks indicate RGCs. *n* = 3 independent experiments. **F** Representative extracted eyes were kept ie immunofluorescence for CTB-488. *n* = 5 independent experiments. **G** Quantification of the mean fluorescence intensity of CTB-488. **H** Representative immunofluorescence for L-glut. Eyes were processed for immunofluorescence with a L-glut-specific antibody (green). *n* = 3 independent experiments. **I** Quantification of the mean fluorescence intensity of L-glut. **Immunofluorescence caption:** OS outer segment, ONL outer nuclear layer, OPL outer plexiform layer, INL inner nuclear layer, IPL inner plexiform layer, GCL ganglion cell layer. 40X magnification. Scale bar 10 μΜ. **Bar plot caption:** Bar representing mean +/− S.D; number of individual replicates per condition shown in the scatter plot. Statistical analysis: One-way ANOVA, two-tailed *post hoc* Tukey test, *p* < 0.05. *p* < 0.05. *p < 0.05; **p < 0.01; ***p < 0.001; ****p < 0.0001.

As the next goal, the protective effect of JGRi1 in the NMDA-induced retinal degeneration model was addressed. To do so, topical treatment with 250 μM of JGRi1, one drop/die, was started one day prior to NMDA injection, then kept on throughout the entirety of the follow-up post-injection. sJGRi1 was included as control (Fig. 7A). Firstly, the protection of JGRi1 on RGC was measured by immunofluorescence for RBPMS and c-CASP3 (Fig. 7B), followed by quantification of RBPMS-positive and c-CASP3-positive cells. Data showed that JGRi1 was able to rescue RGC viability upon NMDA injection (Fig. 7B, C). Conversely, c-CASP3 immunoreactivity, which increased during NMDA injection, was reduced by JGRi1 treatment (Fig. 7B, D). As expected, treatment with sJGRi1 had no effect on either RGC viability or apoptosis.

Next, hematoxylin/Eosin staining was carried out to evaluate the retinal cytoarchitecture (Fig. 7E). NMDA injection produced a disruption of the three nuclear layers of the retina, with a marked effect especially on the INL and GCL, causing nuclei to lose their physiological compactness, as already reported [45, 46]. 250 μM JGRi1 treatment restored tissue architecture only in the GCL, while sJGRi1 failed to do so (Fig. 7E – white asterisks).

The protective effect of JGRi1 on NMDA-induced RGC damage was assessed by uptake and transport of the neural tracer CTB-488. The analysis showed that NMDA alone and with sJGRi1 treatment diminished CTB uptake and transport in RGCs. In contrast, treatment with JGRi1 was able to prevent at least in part this NMDA-induced decrease, suggesting that the peptide preserves the functional integrity and health of RGCs in the presence of excitotoxic stress (Fig. 7F, G).

Lastly, to evaluate L-glutamate levels in retinal tissue from the NMDA-injected eyes, the L-glutamate immunofluorescence was measured. NMDA injection led to a higher L-glut immunoreactivity in the murine retina, consistent with previous data from NMDA-injected rodent models [46–48], especially between the INL and ONL; the treatment with 250 μM JGRi1 reduced NMDA-induced glutamate elevation, even below control levels (Fig. 7H, I). sJGRi1, on the other hand, had no effect.

### JGRi1 reduces the NMDA-induced expression of JNK2 and STX1A and prevents their interaction

**Fig. 8:**
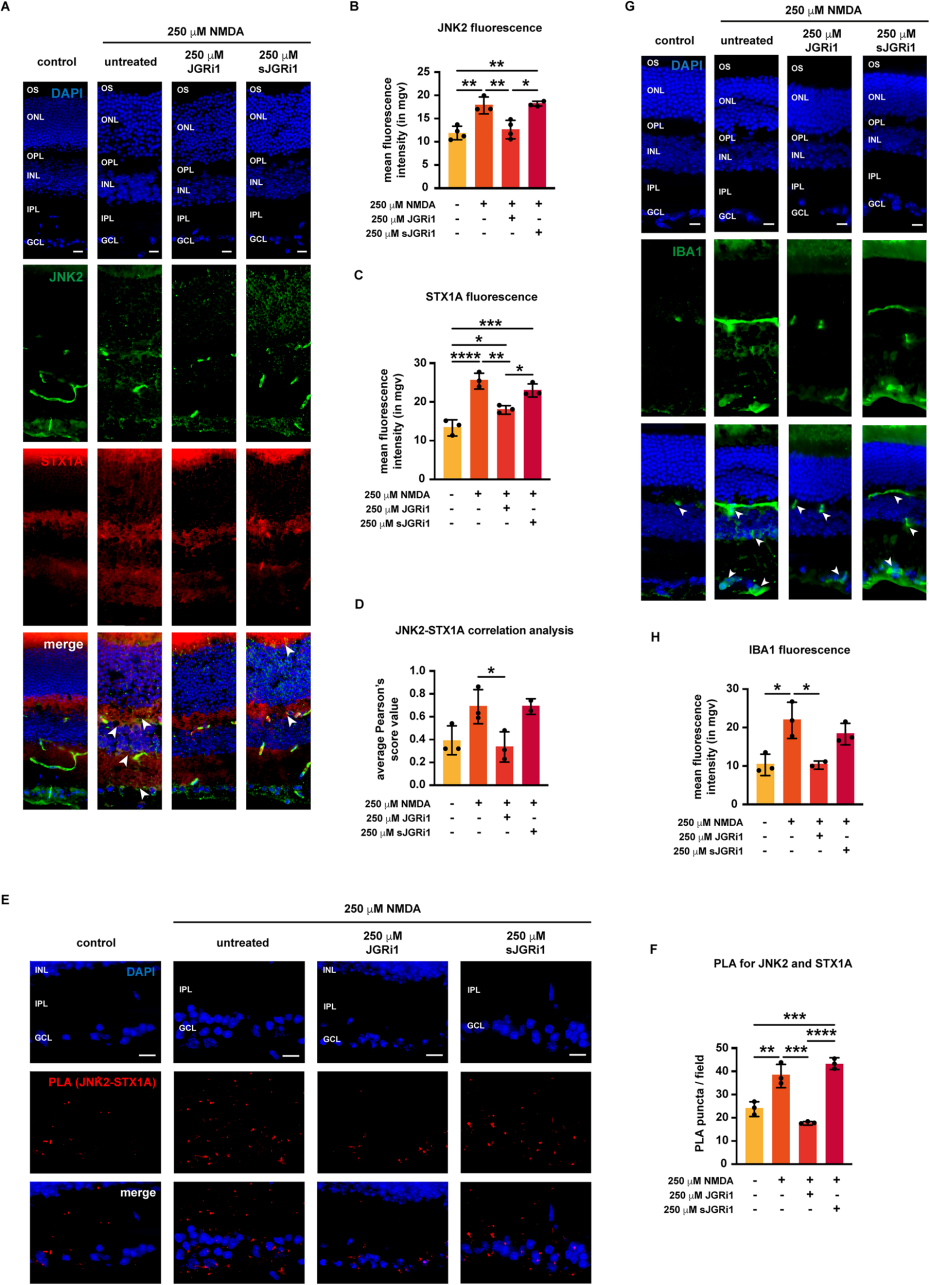
JGRi1 reduces the NMDA-induced expression of JNK2 and STX1A, reduces their interaction and microglial infiltration. **A** Representative co-immunofluorescence for JNK2 and STX1A. Eyes were processed for immunofluorescence with a JNK2-specific antibody (green) and a STX1A specific antibody (red). White arrowheads in the merge column indicate areas where JNK2 and STX1A signals strongly co-localize. *n* = 4 independent experiments. **B** Quantification of the mean fluorescence intensity of JNK2. **C** Quantification of the mean fluorescence intensity of STX1A. **D** Correlation analysis between JNK2 and STX1A signals. Pearson’s scores were calculated for each image from (**A**). **E** Proximity ligation assay (PLA) for JNK2 and STX1A. Eyes were processed for PLA according to manufacturer’s instructions. Red puncta indicate JNK2-STX1A interactions. *n* = 4 independent experiments. **F** Quantification of PLA puncta per field. **G** Representative immunofluorescence for IBA1. Eyes were processed for immunofluorescence with a IBA1-specific antibody (green). White arrowheads in the merge column indicate areas strongly immunoreactive to IBA1. *n* = 5 independent experiments. **H** Quantification of the mean fluorescence intensity of IBA1. **Immunofluorescence caption:** OS outer segment, ONL outer nuclear layer, OPL outer plexiform layer, INL inner nuclear layer, IPL inner plexiform layer, GCL ganglion cell layer. 40X magnification. Scale bar 10 μΜ. **Bar plot caption:** Bar representing mean +/− S.D; number of individual replicates per condition shown in the scatter plot. Statistical analysis: One-way ANOVA, two-tailed *post hoc* Tukey test, *p* < 0.05. *p* < 0.05. *p < 0.05; **p < 0.01; ***p < 0.001; ****p < 0.0001.

The behavior of STX1A and JNK2 in response to the different treatments was examined by co-immunofluorescence. As already shown in Fig. 2J, NMDA injection resulted in more JNK2 and STX1A expression in the retina (Fig. 8A). Treatment with JGRi1, but not sJGRi1, reduced JNK2 immunoreactivity in the inner retina, which remained detectable in the GCL and slightly in the ONL as well. As expected, sJGRi1 did not prevent the NMDA-induced increase in JNK2 expression in the inner retina (Fig. 8A, B). Interestingly, unlike what we have seen in the evONC model, here JGRi1 was able to prevent the NMDA-induced increase of STX1A immunoreactivity, which was subsequently confirmed by the quantification of the fluorescence intensity in the retina (Fig. 8A–C). To address the effect of the JGRi1 on the JNK2-STX1A interaction, we employed two different approaches. The first approach consisted of running a correlation analysis between the two fluorescent signals to obtain Pearson’s score. JNK2 and STX1A showed a higher yet not significant Pearson’s score upon NMDA injection, while treatment with JGRi1, but not sJGRi1, led to a significant decrease (Fig. 8D). The second approach consisted in performing proximity ligation assay (PLA), which allows to visualize the interaction between the two proteins as red puncta. In all tested conditions, PLA puncta were detectable mainly in the GCL and in the IPL (Fig. 8E). The number of PLA puncta increased in the slices of NMDA-injected mice, while it was reduced by treatment with JGRi1, but not sJGRi1 (Fig. 8F).

### JGRi1 reduces the NMDA-induced microglial infiltration in the retina

Microglial activation is an important contributor to RGC degeneration in different models of NMDA-induced retinal injury [49, 50]. On this premise, the potential effect of the peptide on microglial reactivity in the retina of NMDA-injected mice was evaluated. Upon NMDA injection, a boost in IBA1 immunoreactivity was noticed throughout the entire retinal tissue, except the ONL (Fig. 8G). Surprisingly, treatment with JGRi1 was able to reduce IBA1 immunoreactivity, although some signal was still detectable in the IPL and the GCL, while no reduction was seen upon treatment with sJGRi1 (Fig. 8G, H).

### JGRi1 protects the INL from NMDA-induced damage and prevents the accumulation of glutamate at synaptic level

The effect of JGRi1 on the viability of A2 amacrine cells and rod bipolar cells was also evaluated in the context of the NMDA injection model. Retinal sections were immunostained for PROX1, and the number of PROX1-positive cells in the INL was counted. As expected, the NMDA injection dramatically decreased PROX1-positive cells in the INL; PROX1-positive cell number was significantly rescued by 250 μM JGRi1, but not sJGRi1 (Fig. S7A, B). Finally, glutamate accumulation at the level of bipolar cell synapses was studied by co-immunostaining for both L-glut and SYT1. The analysis revealed that NMDA injection fostered synaptic glutamate accumulation, which was readily reversed by topical JGRi1, but not sJGRi1, as confirmed by Pearson’s analysis (Fig. S7C, D).

## Discussion

The pursuit of neuroprotection in RDs has been hampered by the intractable challenge of targeting glutamate excitotoxicity without disrupting essential synaptic function. Despite their success in the pre-clinical phase [11–14], the repeated clinical failure of broad NMDAR antagonists like memantine underscores the need for a more precise, targeted strategy [15, 16]. Here, we shift the therapeutic paradigm from postsynaptic receptor blockade to the presynaptic source of excitotoxicity. Previously, we have shown that the disruption of PPI between JNK2 and STX1A by JGRi1 prevents glutamate spillover and grants protection in in vitro models [19, 20]. However, in this work we provide first-time evidence that nPING also represents a valid druggable target and that its disruption constitutes a viable and potent neuroprotective strategy also in vivo RD models. The peptide platform enabled a breakthrough in delivery: topical administration as eye drops, thereby bypassing the systemic limitations that doomed previous oral therapies. Indeed, F-JGRi1 provided evidence that the peptide was able to reach the retina upon both ex vivo and in vivo treatments.

Firstly, we demonstrated that the nPING mechanism is actually involved in RDs. We showed that both in the evONC model and the intravitreal NMDA injection model, there was an upregulation of JNK2 and STX1A. This was associated with an increased interaction between the two proteins, as shown by the PLA and Pearson’s analyses, to a higher and toxic rate of SNARE complex formation and to an enhanced glutamate immunoreactivity in the retina. These findings of higher glutamate levels in the retina upon injury are in line with both our previous high-performance liquid chromatography reports showing an enhancement of retinal glutamate levels upon evONC [25], and with data from rodents intravitreally injected with NMDA [46–48]. Interestingly, our data also show that NMDA alone is less effective than axotomy (with or without NMDA) at inducing JNK2-STX1A interaction. This may indicate that the nPING mechanism may be activated by mechanical injury or stress to RGC axons. This would then “set the stage” for subsequent excitotoxic injury, thus pointing to a potential biphasic mechanism for RGC degeneration, especially in glaucoma. Indeed, we speculate that intraocular pressure elevation in glaucoma may trigger nPING, thus fostering a subsequent excitotoxic damage.

The increase of JNK2 levels is in line with previous reports showing that JNK2 is specifically activated and contributes to RCG death in both a retinal axonal injury-induced model and an intravitreal endotelin-1 injection model [51, 52], although another paper suggested that JNK2 may protect RGC from glaucomatous degeneration in ocular hypertensive DBA/2J mice [53]. Interestingly, to date, there have been no reports about changes in the expression of STX1A levels in specific RDs.

Having established the activation of the nPING pathway in retinal degeneration, we next asked whether its targeted disruption could halt the excitotoxic cascade. JGRi1 treatment did not merely suppress global neurotransmission; it precisely uncoupled the JNK2-STX1A interaction, as evidenced by PLA and Pearson’s analyses. Moreover, since the JNK2 and STX1A interaction is selectively triggered during excitotoxicity, JGRi1 does not interfere with retinal physiology. Such observation can be inferred from the fact that retinas treated with JGRi1 without experiencing any injury retain a normal pattern of expression of both JNK2 and STX1A and, moreover, do not display any change in glutamate immunoreactivity.

In the evONC model, JGRi1 reduced JNK and STX1A phosphorylation, in line with our previous reports [19, 20]. Worth of notice, JNK2 expression levels remained significantly higher than the control upon treatment with all tested dosages of JGRi1. Such a result may fit with the idea that JNK2 may protect RGC from degeneration to some extent, in line with previous reports [53]. The specific disruption of the JNK2-STX1A interaction by JGRi1 subsequently normalized pathogenic SNARE complex assembly and synaptic glutamate levels, effectively breaking the self-amplifying cycle of nPING. Moreover, JGRi1 was able to reduce microglial infiltration inside the retinas from NMDA-injected mice. Microglial infiltration has been reported in different models of NMDA-induced retinal injury [49, 50]. A possible explanation for this may lie in the fact that JNK2 promotes microglial activation and the release of pro-inflammatory mediators [54]. Therefore, we speculate that JGRi1, by inhibiting JNK2, may also reduce the degree of microglial infiltration within the tissue.

Treatment with JGRi1 also reduced the NMDA-induced expression of GS, one of the most important enzymes involved in retinal glutamate metabolism, as it is highly expressed in the Müller glia, where it converts glutamate to glutamine [31]. Our funding of an NMDA-induced expression of GS may seem quite in contrast with what has already been reported, as GS levels were found to be reduced in the retina of patients suffering from geographic atrophy [55]. However, a previous paper showed that after retinal ischemia, GS can be upregulated for a short period of time before its levels start to decay. Therefore, since our retinal wholemounts were cultured for 24 hours, we believe that the higher GS levels may represent an early event occurring in order to prevent NMDA-induced damage [56].

JGRi1 showed a protective effect on cells from the INL. This particular layer of the retina hosts multiple cell types, including rod bipolar cells and A2 amacrine cells, which are key interneurons involved in night vision, and they have both been shown to be extremely vulnerable to glaucomatous degeneration [34]. Rod bipolar cell degeneration precedes RGC degeneration in glaucoma models, as it disrupts normal neurotransmission, leading to elevated extracellular glutamate and subsequent excitotoxic damage to RGCs [35]. Our data are in line with this report, as we found that in both the evONC and NMDA injection models, there was a strong co-localization between glutamate and the synaptic marker SYT1 in the IPL, which was counteracted by JGRi1 treatment. Based on this evidence and on the fact that the loss of night vision occurs early in the glaucomatous pathogenesis [40], it is tempting to speculate that an early treatment with JGRi1 could slow down glaucomatous progression by protecting both cells in the INL and the GCL. Indeed, JGRi1 actively reduced RGC degeneration in all tested models. As said, RGCs are particularly vulnerable to glutamate excitotoxicity, although with some differences between the various subtypes [57]. Our data clearly show that JGRi1 can preserve RGC function and viability, as shown by the increase in RGC marker expression found in all our models and the restored CTB-488 tracking. Such evidence is particularly important for retinal protection purposes, as RGC degeneration is a hallmark not only of glaucoma, but also of several conditions leading to significant visual compromise, such as hereditary optic neuropathies, ischemic optic neuropathies, and demyelinating eye diseases [58].

Worth noting, our data reveal a potent dose-dependent suppression of glutamate release, underscoring the efficacy of JGRi1. The correlation between higher doses, reduced SNARE complex formation, and neuroprotection also defines a critical therapeutic window. Future studies optimizing the dosing regimen will be essential to fine-tune the inhibition of pathological glutamate spillover while preserving essential physiological neurotransmission, a key step in translational development.

In summary, we have moved beyond the failed paradigm of NMDAR blockade by identifying and targeting a presynaptic excitotoxic hub. Our work provides two foundational advances: first, it establishes the nPING pathway as a critical driver of retinal degeneration and a druggable target; second, it introduces a novel first-in-class therapeutic modality that offers a direct and clinically viable path to neuroprotection. This strategy, focused on precision and localized delivery, opens a new front in the fight against not only retinal diseases but potentially a range of CNS disorders driven by excitotoxicity.

## Materials & Methods

### Animals

C57BL/6J mice (male, 25–30 g, 4–5 weeks) were rendered and housed in a temperature- and humidity-controlled condition on a 12:12 light-dark cycle with ad libitum food. The number of animals / experimental group was calculated by G*Power analysis with α = 0.05. Inclusion criteria included: absence of systemic clinical signs (lethargy, significant weight loss, infections); baseline ocular integrity prior to treatment (transparent cornea, absence of corneal lesions/ulcers, absence of cataract or major opacity, absence of evident ocular malformations); treatment compliance. Exclusion criteria included: pre-existing ocular pathology (corneal opacity, ulcers, cataract, microphthalmia); treatment-related ocular adverse events (corneal abrasions, ulceration, edema); inadequate dosing (inappropriate estimated volume delivered, major overflow). No randomization or blinding were performed during experimental group definition.

At the end of treatments, animals were euthanized via beheading or transcranial perfusion with heparin-containing saline followed by 4% paraformaldehyde. Furthermore, since the glutamate synthesis and release are circadian-dependent, animals from all experimental groups were sacrificed at the same daytime.

### Ex vivo permeability assay

To assess JGRi1 permeability ex vivo, eyeballs from euthanized animals were collected and immersed into a balanced salt solution (BSS) buffer (137 mM NaCl, 5.4 mM KCl, 1.8 mM CaCl_2_•2H_2_O, 0.98 mM MgCl_2_•6H_2_O, 8.1 mM Na_2_HPO_4_•2H_2_O, 1 mM K_2_HPO_4_, 5.5 mM D-glucose, 4.2 mM NaHCO_3_, pH 7.4), then incubated for 1 hour at 37 °C with different concentrations (50 μM, 250 μM, 500 μM) of a fluorescently labeled version of our JGRi1 peptide (F-JGRi1). A version of F-JGRi1 devoid of the Tat sequence, which grants its cellular permeability (ΔTat-F-JGRi1), was included as an additional control. Afterwards, eyeballs were washed three times with BSS to remove excess of the peptide, then processed according to experimental needs.

### In vivo permeability assay

For the in vivo permeability assay of JGRi1, animals were treated with a single drop per day of F-JGRi1 at different concentrations (50 μM, 250 μM, 500 μM) dissolved in BSS buffer. A total volume of 5 μL of JGRi1 solution was applied directly onto the cornea. Treated animals were kept immobilized and under observation for 1 minute to ensure proper peptide penetration within the eye. At the end of treatments, animals were sacrificed, eyeballs were collected and processed accordingly. ΔTat-F-JGRi1 was also included as an additional control.

### Ex vivo optic nerve cut (evONC)

Mice were either left untreated or treated for 6 days with a single drop per day of JGRi1 dissolved into a balanced saline solution (BSS) buffer at different concentrations (50 μM, 250 μM, 500 μM). A total volume of 5 μL of JGRi1 solution was applied directly onto the cornea. Treated animals were kept immobilized and under observation for 1 minute to ensure proper peptide penetration within the eye. Animals were then euthanized via beheading. A scrambled version of the peptide (sJGRi1) at a concentration of 500 μM was included as a control. Subsequently, the optic nerve was cut, and the eyes were harvested according to our previously established protocol [25, 26]. The extracted eyes were kept in phosphate-buffered salt (PBS; Capricorn Scientifics, Germany) at 4 °C for 24 hours, then the retinas were dissected and processed accordingly.

### Peptide design and synthesis

The identification and production of the amino acid sequence of JGRi1 have already been described [19]. Briefly, docking simulations between JNK2 and STX1A were performed by running the Rosetta 3.427 software and ranked based on the total score of the interaction, allowing the identification of the ‘minimal contact area’ between JNK2 and the N-terminal portion of STX1A. Based on computational output, a small peptide was designed to disrupt the JNK2-STX1A interaction. The effector sequence (IEQSIEQEEGLNRS), which is part of the N-terminal sequence of STX1A corresponding to the minimal contact area with JNK2, was attached to 12 residues (GRKKRRQRRRPP) corresponding to the HIV-1 Tat protein, to confer cell permeability. F-JGRi1 was produced by adding fluorescein isothiocyanate to the N-terminal portion of the protein. ΔTat-F-JGRi1 was obtained by removing the Tat sequence and by linking FITC to the N-terminal portion of the peptide. sJGRi1 was obtained by scrambling up the effector sequence of the peptide (RISEQLSNIEEGQE), without modifying the Tat sequence to retain cell permeability.

The peptides were synthesized by microwave-assisted solid phase synthesis [59] based on 9-Fluorenylmethyloxycarbonyl (Fmoc) chemistry on pre-loaded Wang resin (0.4 meq/g substitution), with a five-fold molar excess of 0.2 M fluorenyl methoxycarbonyl-protected amino acids dissolved in N-methyl pyrrolidinone, and using HOBT/HBTU/DIEA (5: 5: 10 eq) as activators.

### Retinal wholemounts

Eye bulbs from C57BL/6 J mice were explanted and put into a Petri dish in the presence of DMEM without fetal bovine serum (FBS) supplementation. Using a stereomicroscope placed under a cell-culture hood, retinas were isolated using surgical forceps, then cut into quarters using surgical scissors, starting from the retinal periphery all the way down to the optic nerve emergence. Afterwards, explants were cultured in 6-well plates containing either fixed or removable inserts (Euroclone, ET3006). 1 mL of DMEM, supplemented with 1:100 penicillin/streptomycin (Lonza, Switzerland) and 10% v/v FBS (Capricorn Scientific, Germany) was added to the well, underneath the insert. Explants were placed on the insert with the ganglion cell layer facing upward, in order to improve photoreceptor survival through contact with the medium beneath the insert. To maintain hydration, an additional 100 μL of medium was applied directly onto the insert, ensuring that the explant remained moist without floating. Afterwards, retinas were incubated for 1 hour at 37 °C, treated with 100 μM NMDA (Merck, Germany) for 2 hours, then reverted to control medium. At 24 hours post-mounting, retinas were processed accordingly. JGRi1 or sJGRi1 at the concentration of 1 μM were applied 1 hour prior to NMDA treatment.

### NMDA injection model

Retinal excitotoxicity was induced unilaterally in adult male C57BL/6J mice (*n* = 18) by intravitreal injection (2 μL) of 250 μM NMDA. Mice were anesthetized using isoflurane gas at 4–5% for induction in an enclosed chamber, then maintained at 2–3% via nose cone during the procedure. Prior to injection, a few drops of proparacaine (0.5% solution) were applied to both eyes to provide local numbing. Unilateral intravitreal injections of NMDA were to the left eye, and an equivalent volume of saline (control) was injected into the vitreous cavity of the mouse using a 33-gauge needle attached to a Hamilton syringe. To minimize the risk of postoperative infection, Tobramycin Ophthalmic Solution (0.3%) was applied topically to the cornea. For pharmacological treatment, mice were randomly assigned to one of three groups: active peptide treatment (to allow protein solubilization = 6), scrambled peptide control (*n* = 6), or an untreated control (*n* = 6). Mice were treated with either an active form of JGRi1 or sJGRi1. Mice were treated daily with 4 µL of 250 µM JGRi1 or 250 µM sJGRi1 applied directly to the cornea as a topical eye drop. Prior to drug treatments, mice were acclimated to scuffing and ocular drop administration. Drug administration began 24 hours prior to NMDA injection and continued throughout the 4-week study period.

Since anterograde axonal transport is a critical indicator of RGC integrity and visual pathway connectivity, we employed Cholera Toxin Subunit B (CTB), a well-established neural tracer that is actively transported along RGC axons. Five days prior to sacrifice, all mice received bilateral intravitreal injections of 1 µL of CTB conjugated to Alexa Fluor 488 (CTB-488) [60–63].

### Preparation of retinal Lysates

After enucleating the eyes, the retinas immediately were extracted and lysated in 100 μl of RIPA buffer (50 mM Tris-HCl pH 7.4, 150 mM NaCl, 1% v/v NP-40, 0.1% w/v sodium-dodecyl-sulfate (SDS), 0.5% w/v sodium deoxycolate, 1 mM ethylenediaminetetraacetic acid (EDTA)) supplemented with a complete set of protease inhibitors (Complete, Roche Diagnostics, Basel, Switzerland) and phosphatase inhibitors (Sigma, St. Louis, MO). Samples were then sonicated, and the homogenates were placed on ice for 30 min to allow protein solubilization. Then, they were centrifuged at 10,000 rpm for 10 min, and subsequently, the supernatant was collected and stored at −80 °C until needed. Samples' concentration was measured by quantifying the A_280_ using a nanodrop system (ThermoFisher).

For retina wholemounts, proteins were extracted using a glass homogenizer containing an extraction solution (50 mM Tris HCl, pH 7.5, Triton X 100%, 20% SDS, 0.5 M EDTA), supplemented with a protease and phosphatase inhibitor cocktail (Thermo Scientific, USA). The retinas were left on ice for 20 minutes to allow the extraction solution to act, and finally centrifuged at 13,200 rpm at 4 °C. Subsequently, the supernatant containing the proteins was collected.

### Western blot

For each sample, 50 μg of protein extract supplemented with 1X Laemmli buffer with the addition of 2.5% β-mercaptoethanol was boiled at 95 °C for 5 minutes, then separated on 10–15% SDS polyacrylamide gel electrophoresis (SDS-PAGE) and transferred to PVDF membranes. Afterwards, the membranes were blocked in 5% non-fat milk powder dissolved in Tris-buffered saline (TBS) supplemented with 0.1% v/v Tween 20 (TBS-T) for 1 hour at room temperature. Afterwards, membranes were incubated overnight at 4 °C with the following primary antibodies diluted either in the same blocking solution or 3% bovine serum albumin (BSA) dissolved in TBS-T: anti-RBPMS (ab15210, Abcam, UK); anti-c-CASP3 (9661, Cell Signaling, USA); anti-JNK2 (sc-271133, Santa Cruz, USA); anti-STX1A (S0664, Merck, Germany); anti-p-STX1A (Ser 14) (CSB-PA053965, Cusabio, USA); anti-STX1A (110302, Synaptic System, Germany); anti-p-JNK (9251, Cell Signaling, USA); anti-JNK (9252, Cell Signaling, USA); anti-BRN3A (sc-8429, Santa Cruz, USA); anti-GS (ab64613, Abcam, UK). To develop the blots, horseradish peroxidase-conjugated secondary antibodies (anti-mouse or anti-rabbit, 1: 10,000, Bio-Rad, USA) were utilized, and the immunoreactive bands were visualized by exposure to the ECL chemiluminescence system (Merck, Germany). Either β-tubulin (ab18207, Abcam, UK) or α-actin (sc-1616, Santa Cruz, USA) was used as the loading control for quantification. Western blots were quantified by densitometry using ImageJ software. Protein expression was expressed as fold-change and obtained by dividing the band density of the sample by that of the loading control. The activation of both JNK and STX1A was expressed as the ratio of the band intensity of phosphorylated form to the total protein.

### SNARE complex assay

The modulation SNARE complex assembly by JGRi1 was evaluated via native PAGE. Briefly, samples were loaded using a non-denaturizing loading buffer (0.5 M Tris-HCl, pH 6.8, 15% v/v glycerol, and 1% v/v bromophenol blue). Samples were then left non-boiled to preserve protein-protein interaction, separated onto polyacrylamide gels, and then blotted onto nitrocellulose membranes. The immunoreactive bands were identified by using an antibody against STX1A, which is a core component of SNARE complexes. Bands starting from approximately 66 kDa were quantified as an indicator of the SNARE complex assembly upon treatment. β-tubulin was used as the loading control for quantification. The rate of SNARE complex formation was expressed as a fold-change and obtained by dividing the band density of the sample by that of the loading control.

### Hematoxylin/Eosin staining

The enucleated eyes were fixed in 4% paraformaldehyde solution overnight at 4 °C. Then, they were incubated in a 30% sucrose-in-PBS solution overnight, and finally, they were embedded in an optimal cutting temperature (OCT, Sigma, St. Louis, MO, USA) compound. Eyes were cut at a thickness of 20 μm. For reliability, the sections containing the optic nerve were utilized, and in each eye, at least five discontinuous sections were analyzed. For histological examination, cryosections were stained in Mayer’s Hematoxylin Solution (Merck, Germany), rinsed in tap water, then counterstained using Eosin Y alcoholic solution (Merck, Germany). Sections were mounted on coverslips with Eukitt (Merck, Germany), and observed under a light-transmission microscope (Nikon) with 40X magnification.

### Immunofluorescence

Retinal cryosections were permeabilized using 0.5% Tryton-X (Merck, Germany) in PBS (Capricorn Scientifics, Germany) solution for 5 minutes. Then, blocked (0.1 M glycine, 2% w/v bovine serum albumin, 2% v/v fetal calf serum, 0.1% v/v Tryton-X) for 1 hour to avoid non-specific protein interactions. The primary antibody was diluted in the same blocking solution and held overnight at 4 °C. The following primary antibodies were used: anti-RBPMS (ab15210, Abcam, UK); anti-c-CASP3 (9661, Cell Signaling, USA); anti-L-Glutamate antibody (ab9440, Abcam, UK); anti-JNK2 (sc-827, Santa Cruz, US and GTX107477, GeneTex, USA); anti-STX1A (S0664, Merck, Germany); anti-STX1A (110302, Synaptic System, Germany); anti-IBA1 (019-19741, Fujifilm Wako, Japan); anti-PROX1 (11067-2-AP, ProteinTech, USA); anti-SYT1 (sc-136480, Santa Cruz, USA). The secondary antibodies were Alexa Fluor® 488 and Alexa Fluor® 594 conjugated-anti-rabbit or anti-mouse IgG (Invitrogen, USA), dissolved in the same blocking solution at a 1:500 dilution for 1 hour. Slides were subsequently mounted on coverslips using a DAPI-containing mounting medium (Fluoromount, Invitrogen, USA), and fluorescent images were acquired using a confocal laser scanning microscope at a 40X magnification (Olympus; Confocal System FV500). Fluorescence intensity was quantified by using ImageJ software. Fluorescence intensity was expressed as mean gray values (mgv) minus background signal calculated in at least 5 different fields per condition. To calculate the number of RBPMS and c-CASP3-positive cells in the GCL, the total number of positive cells was divided by the total number of cells in the GCL, then converted in percentage. At least 100 cells per condition were used for the analysis.

### Quantification of F-JGRi1 signal

Retinal cryosections were mounted on coverslips using a DAPI-containing mounting medium (Fluoromount, Invitrogen, US), and fluorescent images were acquired using a confocal laser scanning microscope at a 40X magnification (Olympus; Confocal System FV500). To measure F-JGRi1 signal intensity in single retinal layers, each layer was selected, and fluorescence in that given layer was quantified by ImageJ. Fluorescence intensity was expressed as mean gray values (mgv) minus background signal, calculated in at least 10 different fields per condition. To calculate the number of FITC-positive cells in the GCL, the total number of FITC-positive cells was divided by the total number of cells in the GCL, then converted in percentage. At least 100 cells per condition were used for the analysis.

### Quantification of CTB-488 signal intensity

Retinal cryosections were mounted on coverslips using a DAPI-containing mounting medium (Fluoromount, Invitrogen, US), and fluorescent images were acquired using a confocal laser scanning microscope at a 40X magnification (Olympus; Confocal System FV500). Fluorescence intensity was quantified by using ImageJ software. Fluorescence intensity was expressed as mean gray values (mgv) minus background signal calculated in at least 5 different fields per condition.

### Proximity Ligation Assay (PLA)

PLA reaction was carried out according to the manufacturer’s instructions (Merck, Germany). Briefly, samples were incubated with primary antibodies overnight at 4 °C and the day after with the PLA PLUS and MINUS probes for 1 hour at 37 °C. All antibodies and probes were diluted in the buffers provided in the kit. Subsequently, probe ligation was carried out by incubating samples for 30 minutes at 37 °C, followed by an amplification step done by incubating samples for 100 minutes at 37 °C. Slides were then mounted on coverslips using the DAPI-containing mounting medium (Merck, Germany). Fluorescent images were visualized by confocal microscopy at a wavelength of 594 nm with 60X magnification. The number of PLA puncta was calculated using ImageJ software. The number of puncta per field was obtained by dividing the total number of counted PLA puncta per the number of field (*n* = 10) used for the analysis.

### Pearson’s co-localization analysis

Co-localization between fluorescent signals in acquired images was expressed using Pearson’s score, which describes the correlation of the intensity distribution between two distinct fluorescent signals. The analysis was carried out using the “JACOP” plug-in available for the ImageJ software. At least 10 different fields per condition were utilized for the quantification.

### Statistical Analysis

Statistical analysis was carried out using the Graph Pad Prism 9 program. All data sets were analyzed assuming a normal distribution pattern. Pairwise comparisons were analyzed running a one-tailed unpaired t-test. For multiple comparisons, a One-way ANOVA, followed by two-tailed Tukey’s *post hoc* test was used. Samples were graphed as mean ± SD, with at least a statistical significance given at *p* < 0.05.

### Cartoons

The cartoons shown in this paper were made using the open-source version of Biorender.

## Supplementary information

Blots uncropped file

Supplementary figure legends

Figure S1

Figure S2

Figure S3

Figure S4

Figure S5

Figure S6

Figure S7

## Data Availability

Data will be made available on request.
