# Supplementary material for: Topical eye treatment with JGRi1, a protein/protein interaction inhibitor, mitigates retinal degeneration
Source: Cell Death Dis. 2026 Apr 15;17(1):504. doi: 10.1038/s41419-026-08717-x (PMC13194685; doi:10.1038/s41419-026-08717-x)
Supplement: Supplementary file 1 — Blots uncropped file [file 41419_2026_8717_MOESM1_ESM.pdf]

Fig. 1L

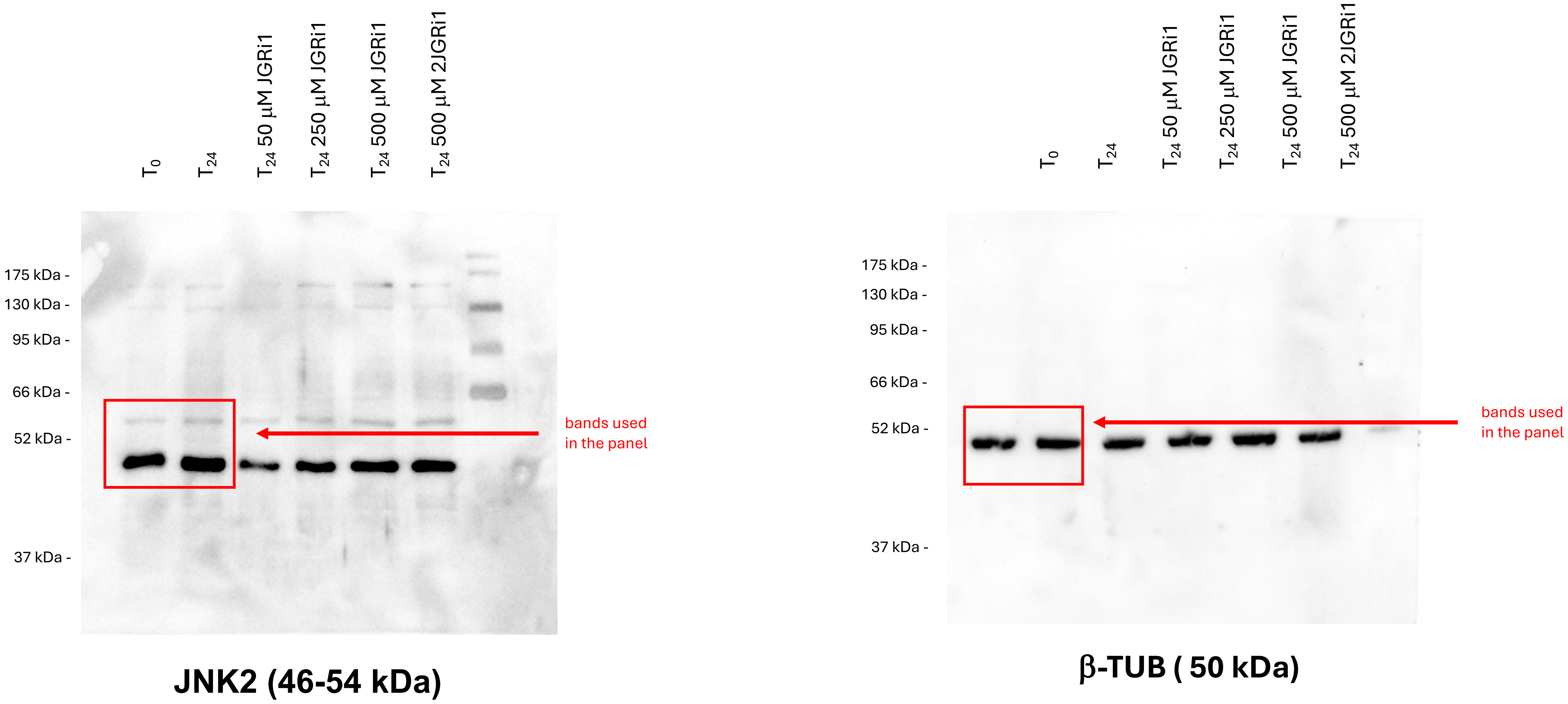

T_0
T_{24}
T_{24} 50 µM JGRi1
T_{24} 250 µM JGRi1
T_{24} 500 µM JGRi1
T_{24} 500 µM 2JGRi1
175 kDa -
130 kDa -
95 kDa -
66 kDa -
52 kDa -
37 kDa -
bands used in the panel
JNK2 (46-54 kDa)
T_0
T_{24}
T_{24} 50 µM JGRi1
T_{24} 250 µM JGRi1
T_{24} 500 µM JGRi1
T_{24} 500 µM 2JGRi1
175 kDa -
130 kDa -
95 kDa -
66 kDa -
52 kDa -
37 kDa -
bands used in the panel
β-TUB (50 kDa)

Fig. 1N

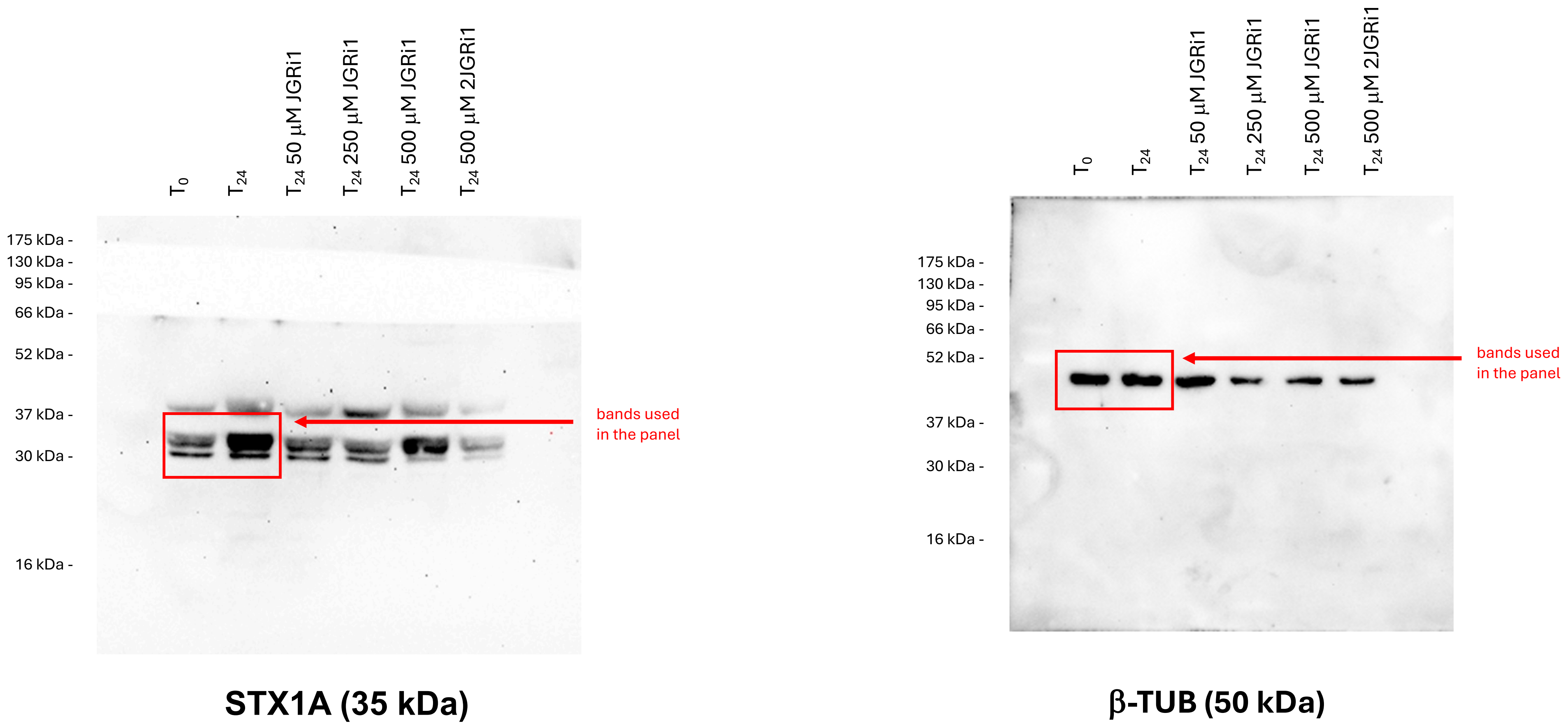
T0
T24
T24 50 µM JGRi1
T24 250 µM JGRi1
T24 500 µM JGRi1
T24 500 µM 2JGRi1
175 kDa -
130 kDa -
95 kDa -
66 kDa -
52 kDa -
37 kDa -
30 kDa -
16 kDa -
bands used
in the panel
STX1A (35 kDa)
T0
T24
T24 50 µM JGRi1
T24 250 µM JGRi1
T24 500 µM JGRi1
T24 500 µM 2JGRi1
175 kDa -
130 kDa -
95 kDa -
66 kDa -
52 kDa -
37 kDa -
30 kDa -
16 kDa -
bands used
in the panel
β-TUB (50 kDa)

Fig. S2C

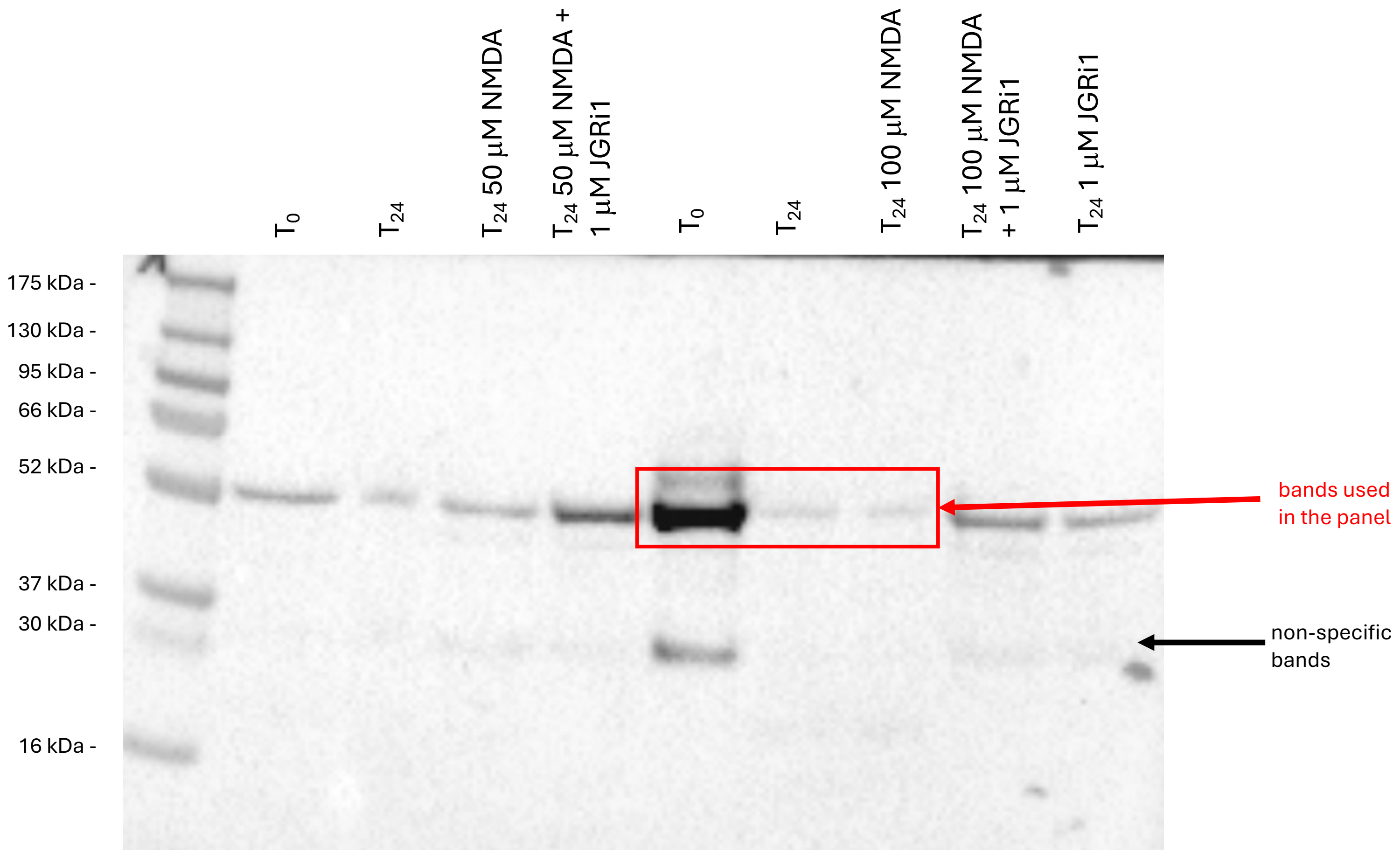

BRN3A (49 kDa)

Fig. S2C

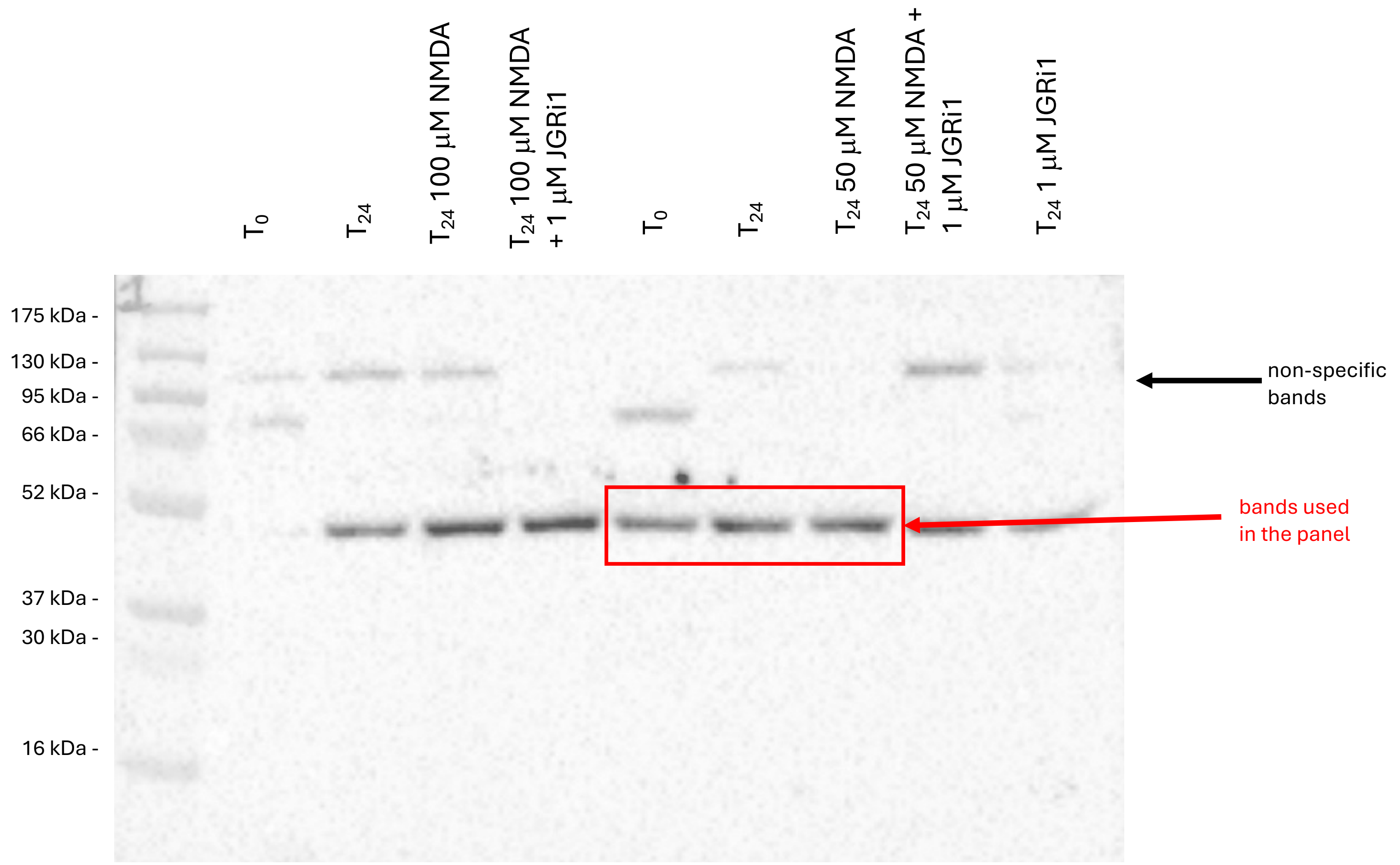

α-actin (42 kDa)

Fig. S2E

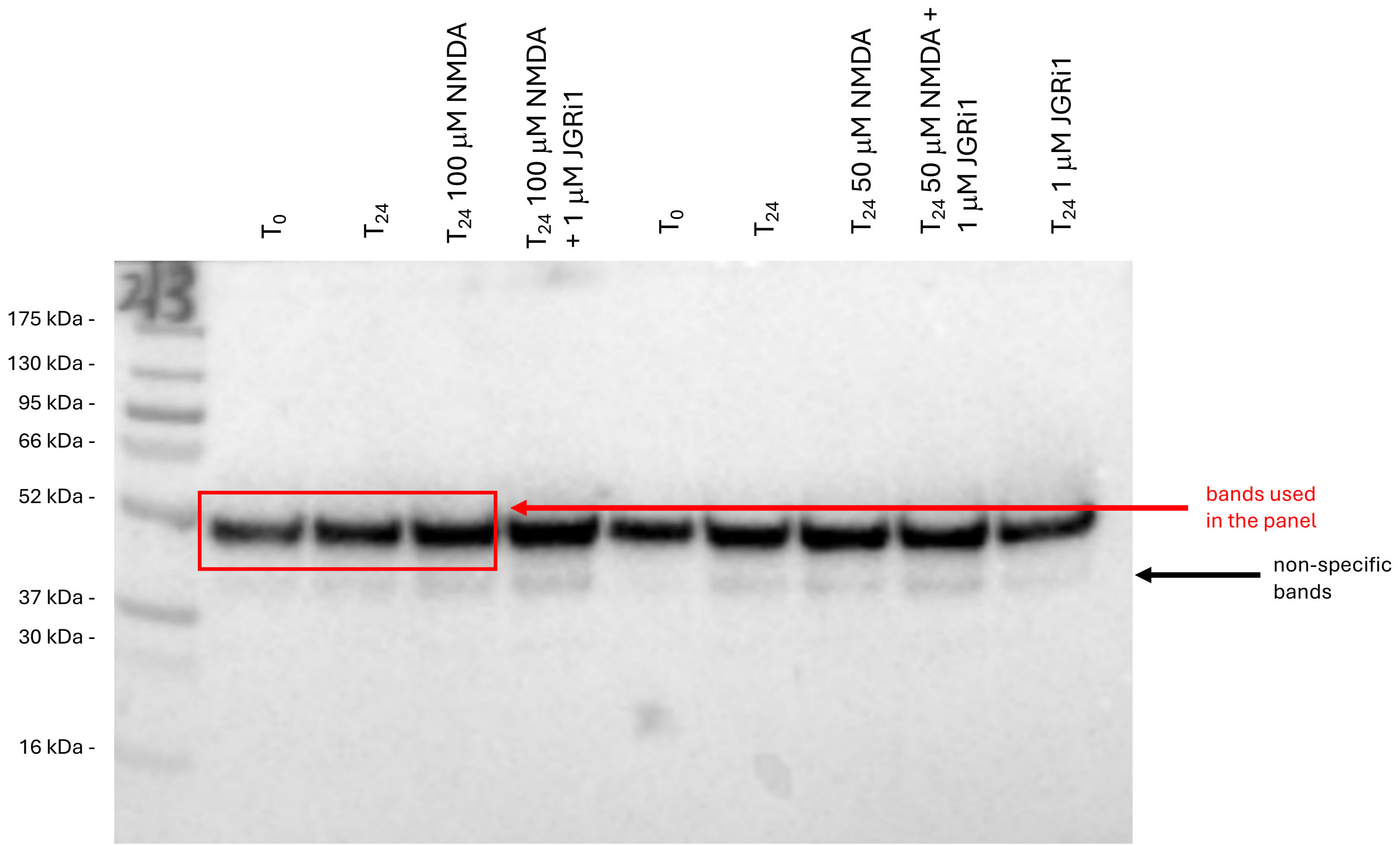

GS (44 kDa)

Fig. S2E

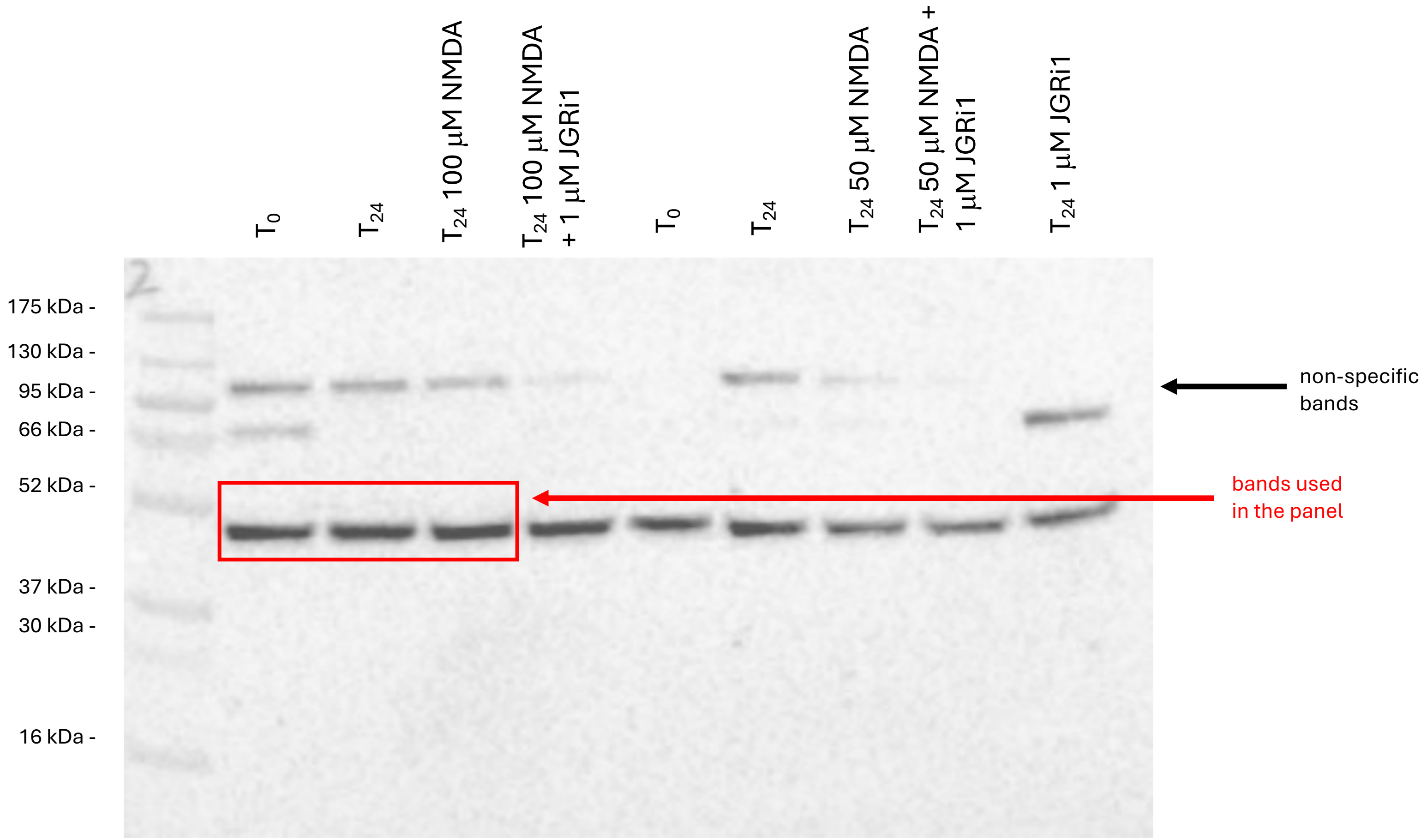

α-actin (42 kDa)

Fig. 4F

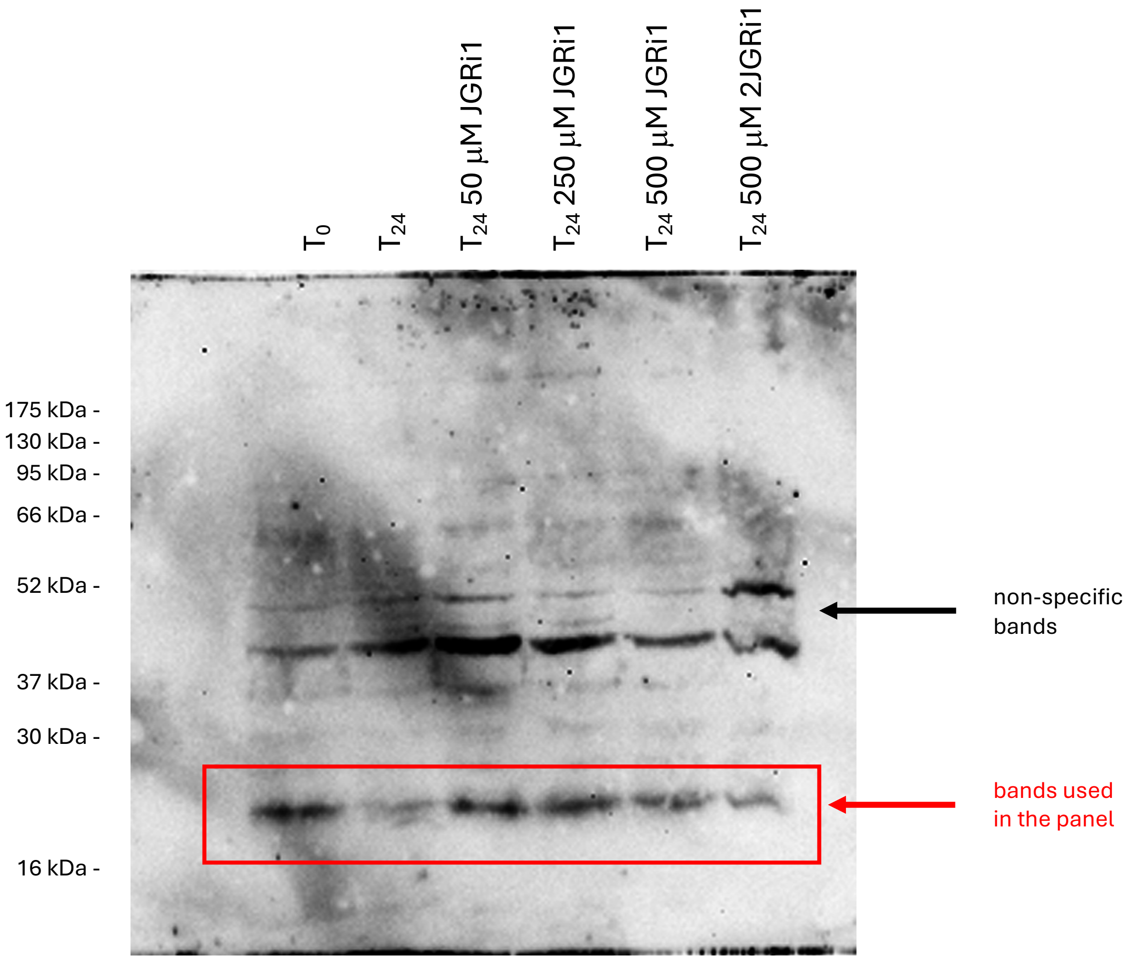

RBPMS (24 kDa)

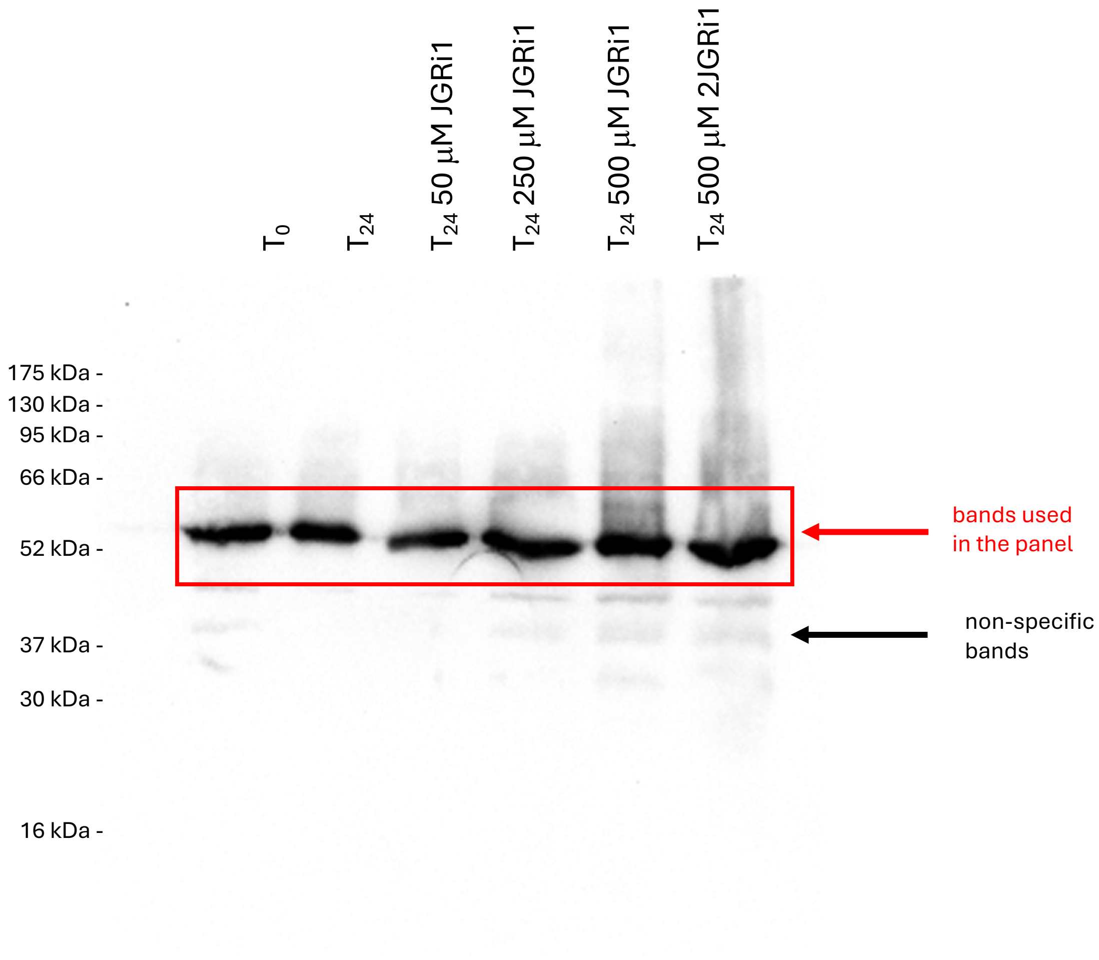

β-TUB (50 kDa)

Fig. 4H

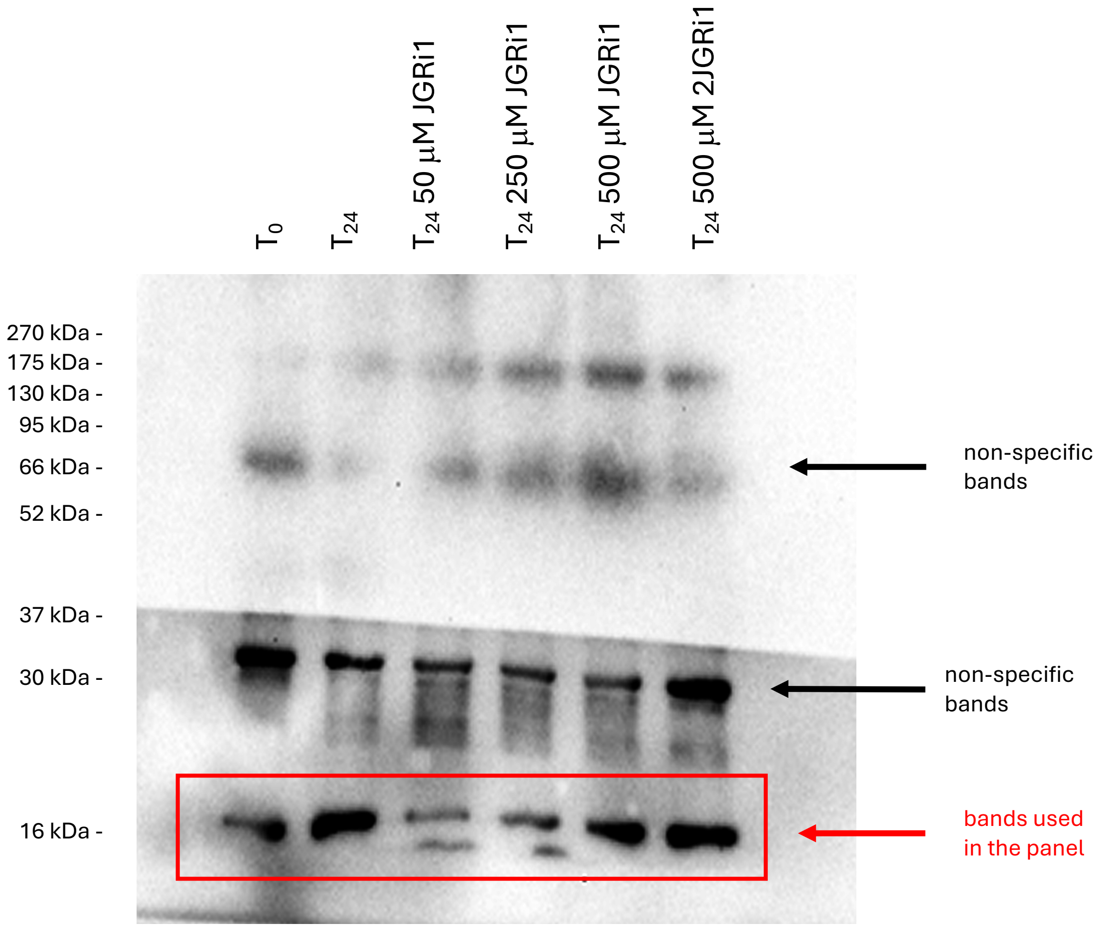

C-CASP3 (17 kDa)

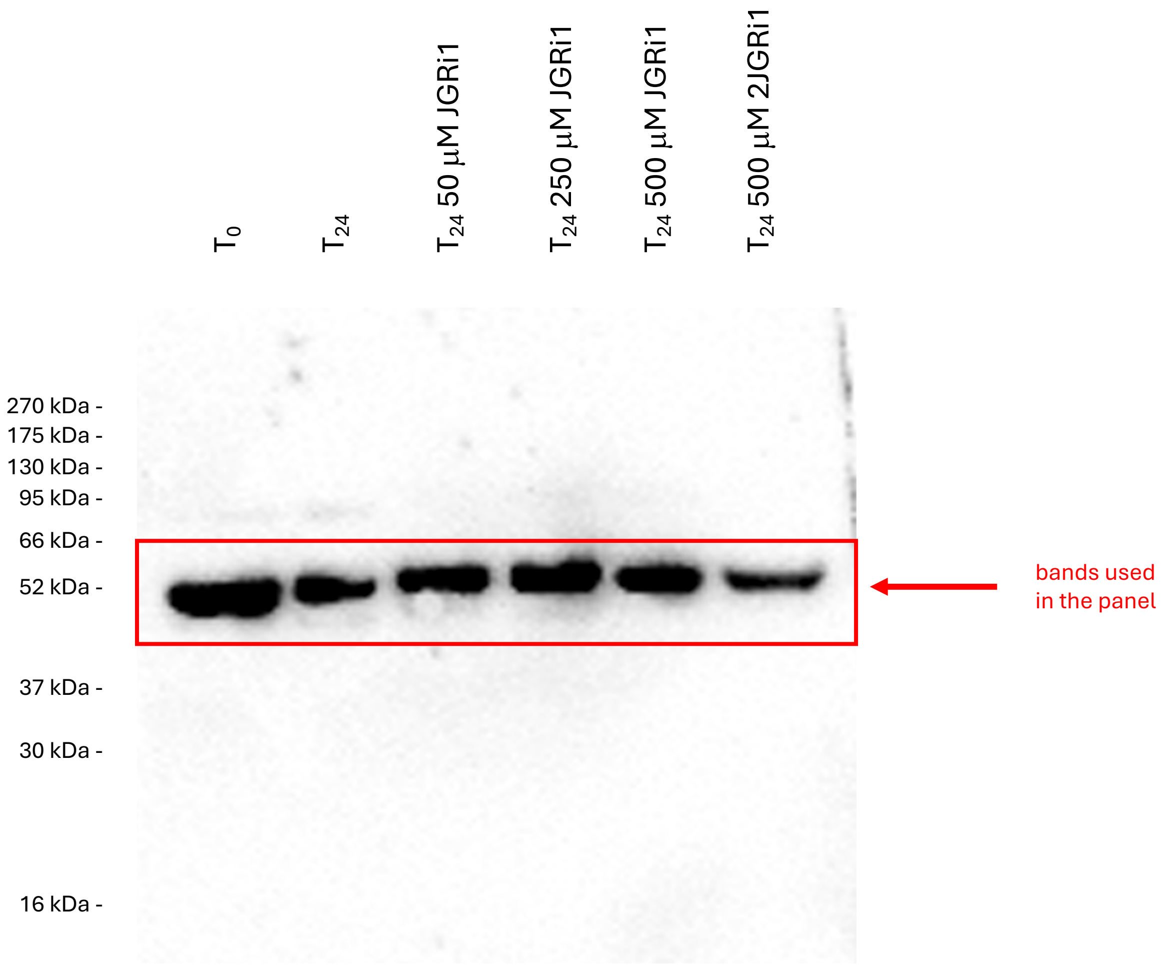

β-TUB (50 kDa)

Fig.5G

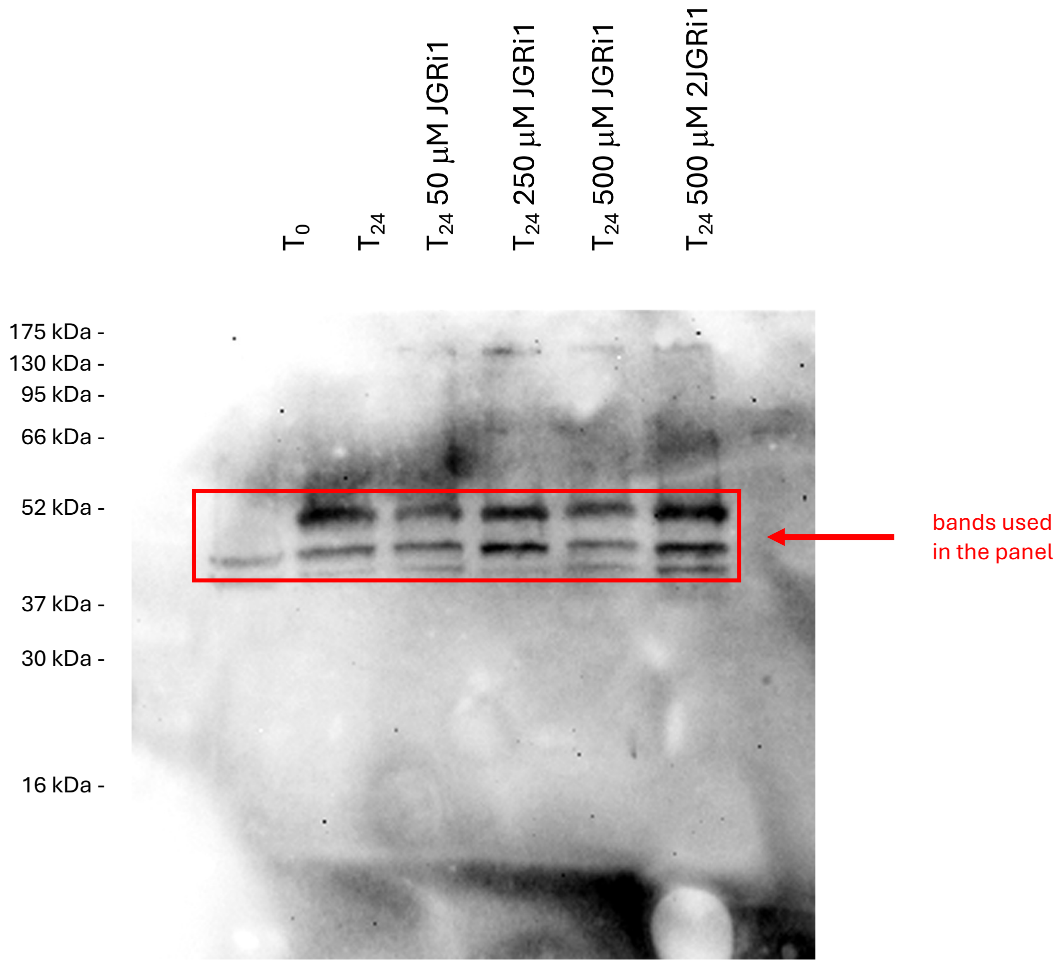

JNK2 (46-54 kDa)

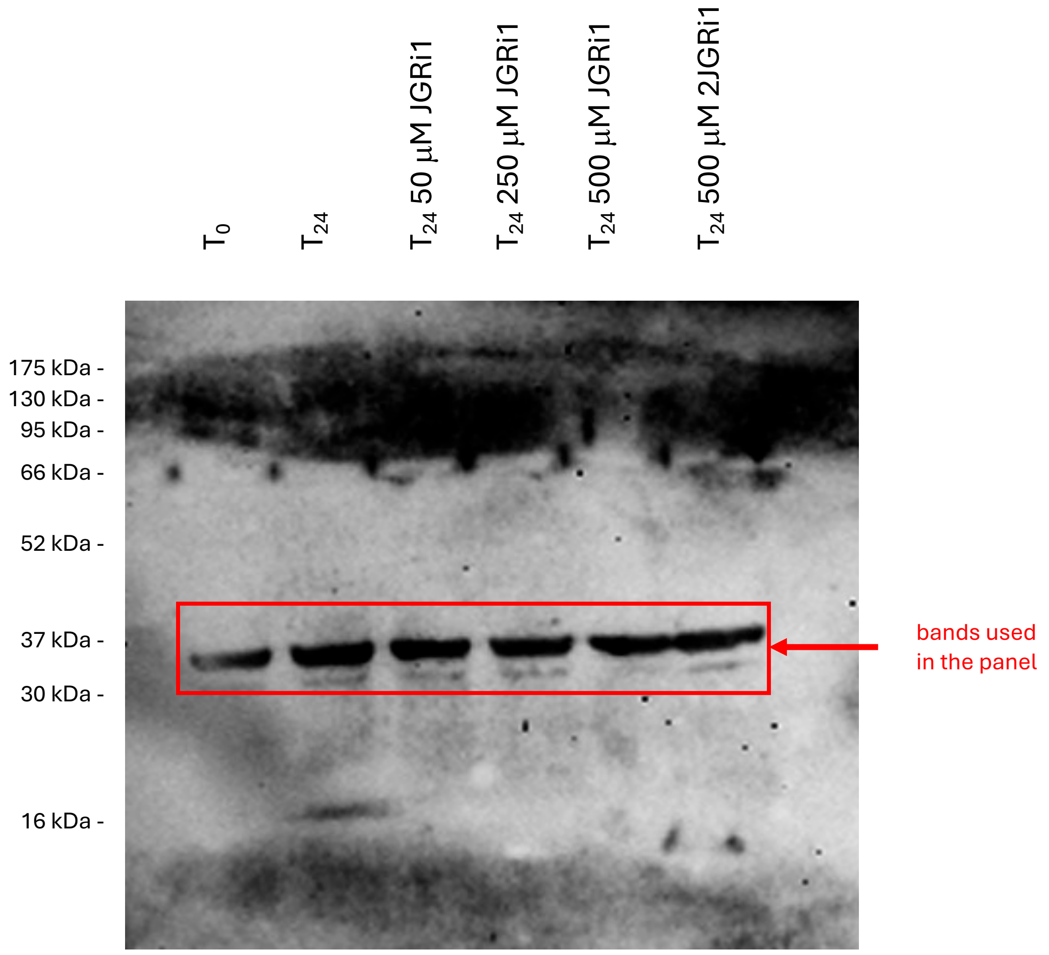

STX1A (35 kDa)

Fig.5G

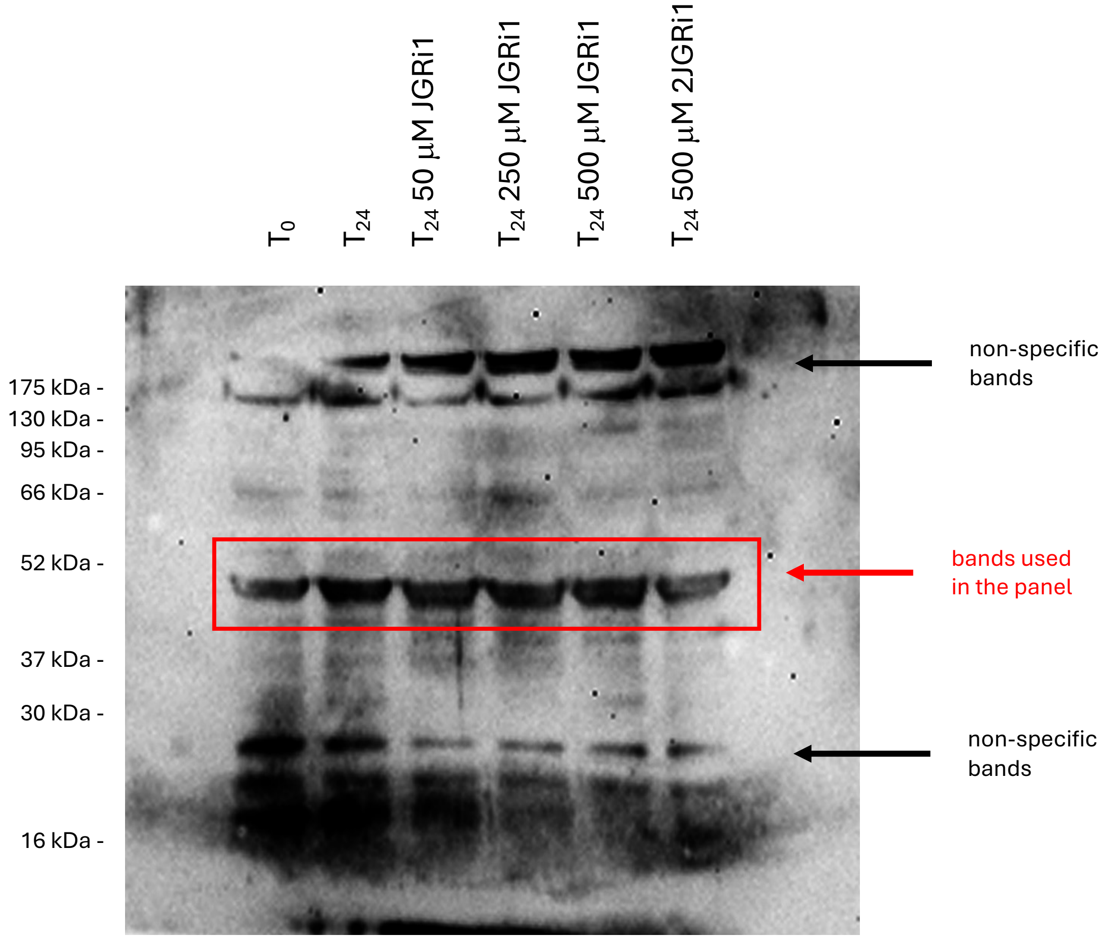

β-TUB (50 kDa)

Fig.6A

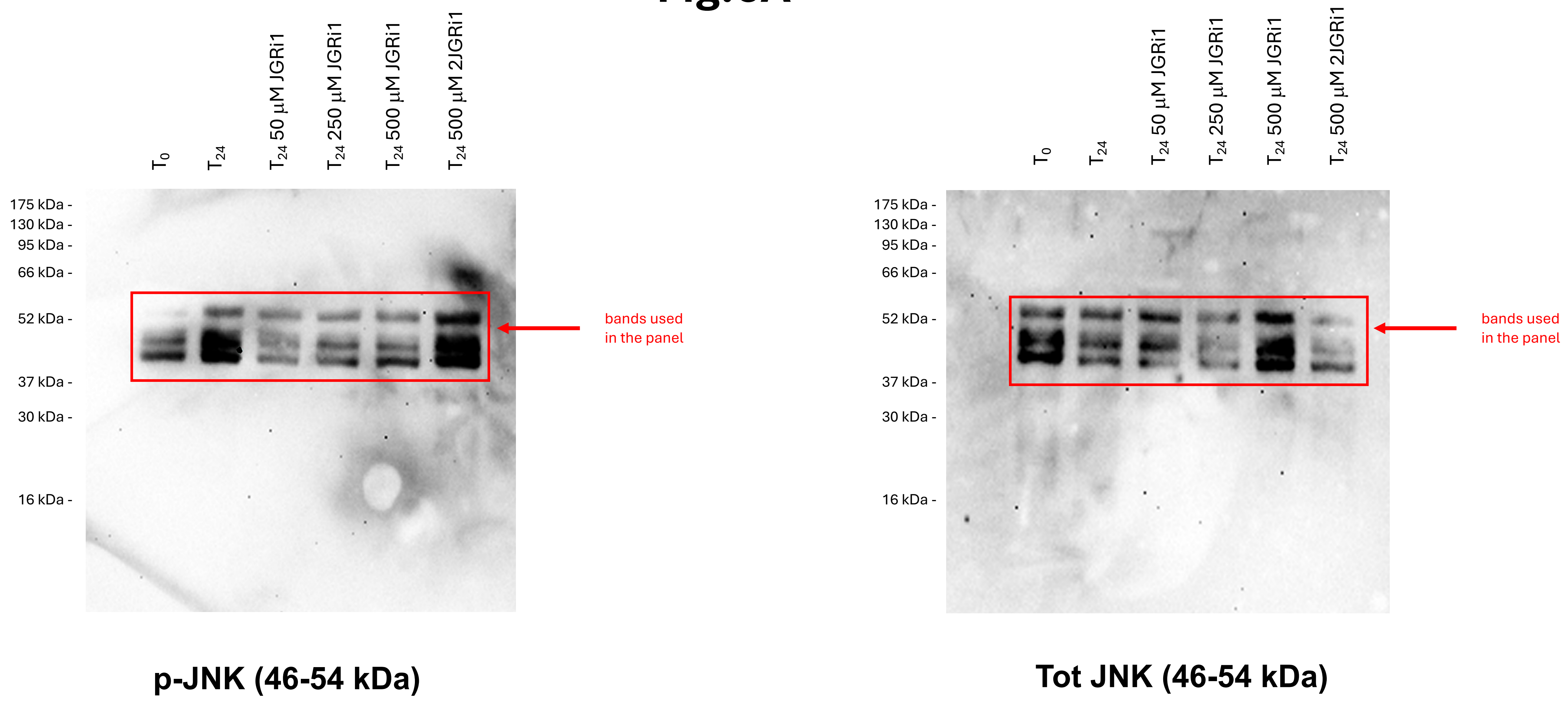
T0
T24
T24 50 µM JGRi1
T24 250 µM JGRi1
T24 500 µM JGRi1
T24 500 µM 2JGRi1
175 kDa -
130 kDa -
95 kDa -
66 kDa -
52 kDa -
37 kDa -
30 kDa -
16 kDa -
bands used in the panel
p-JNK (46-54 kDa)
T0
T24
T24 50 µM JGRi1
T24 250 µM JGRi1
T24 500 µM JGRi1
T24 500 µM 2JGRi1
175 kDa -
130 kDa -
95 kDa -
66 kDa -
52 kDa -
37 kDa -
30 kDa -
16 kDa -
bands used in the panel
Tot JNK (46-54 kDa)

Fig.6A

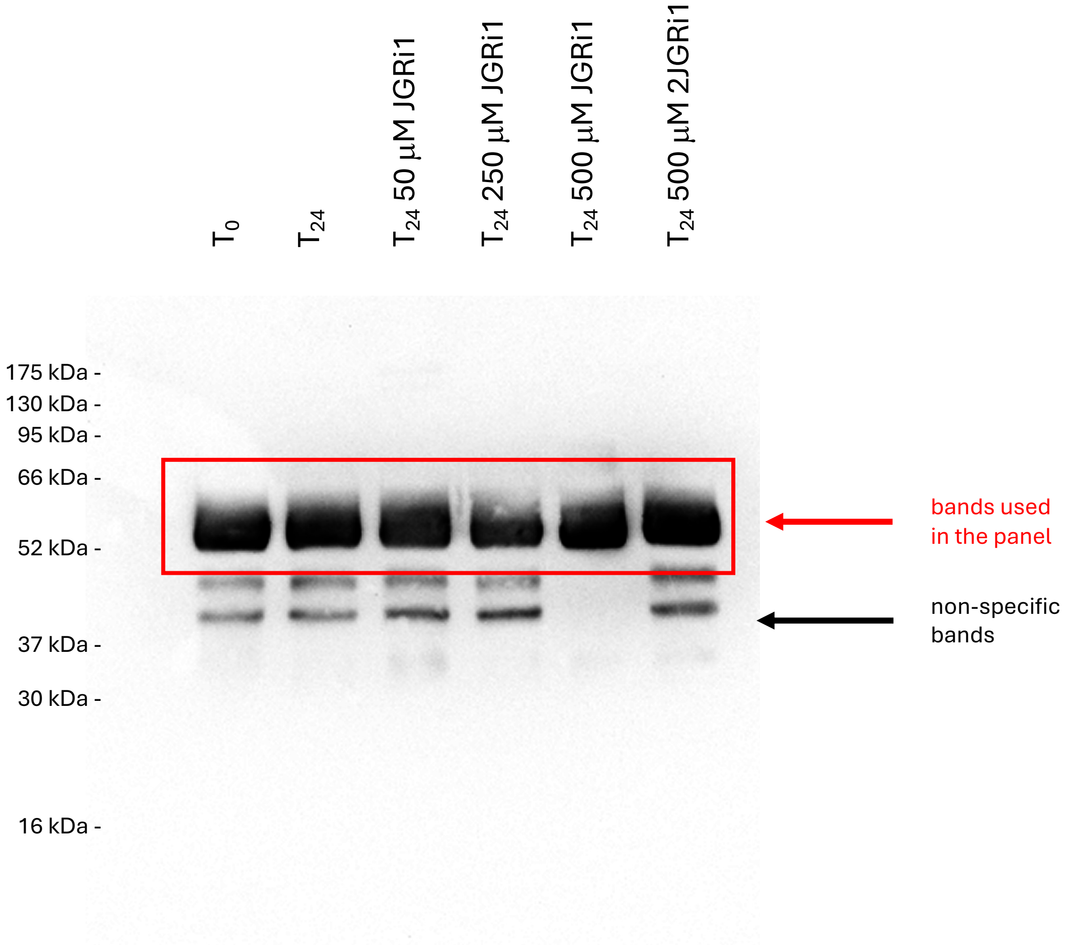

β-TUB (50 kDa)

Fig.6D

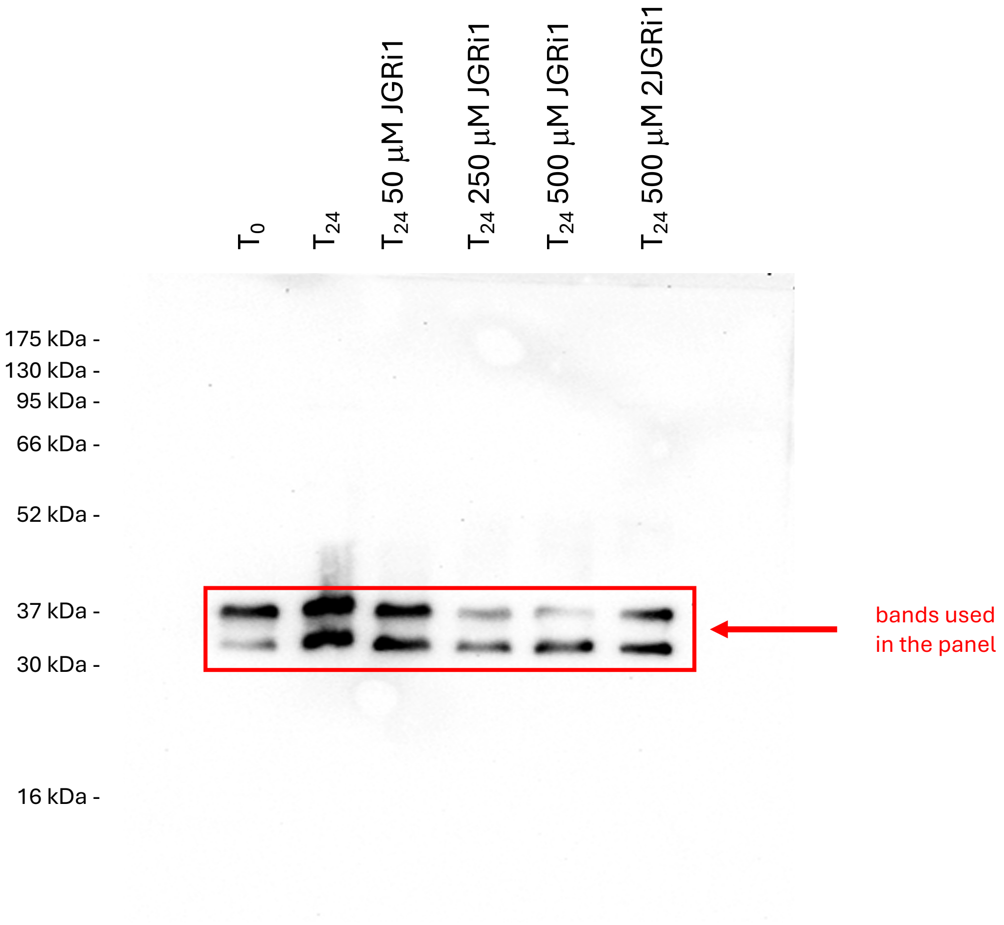

p-STX1A (37-35 kDa)

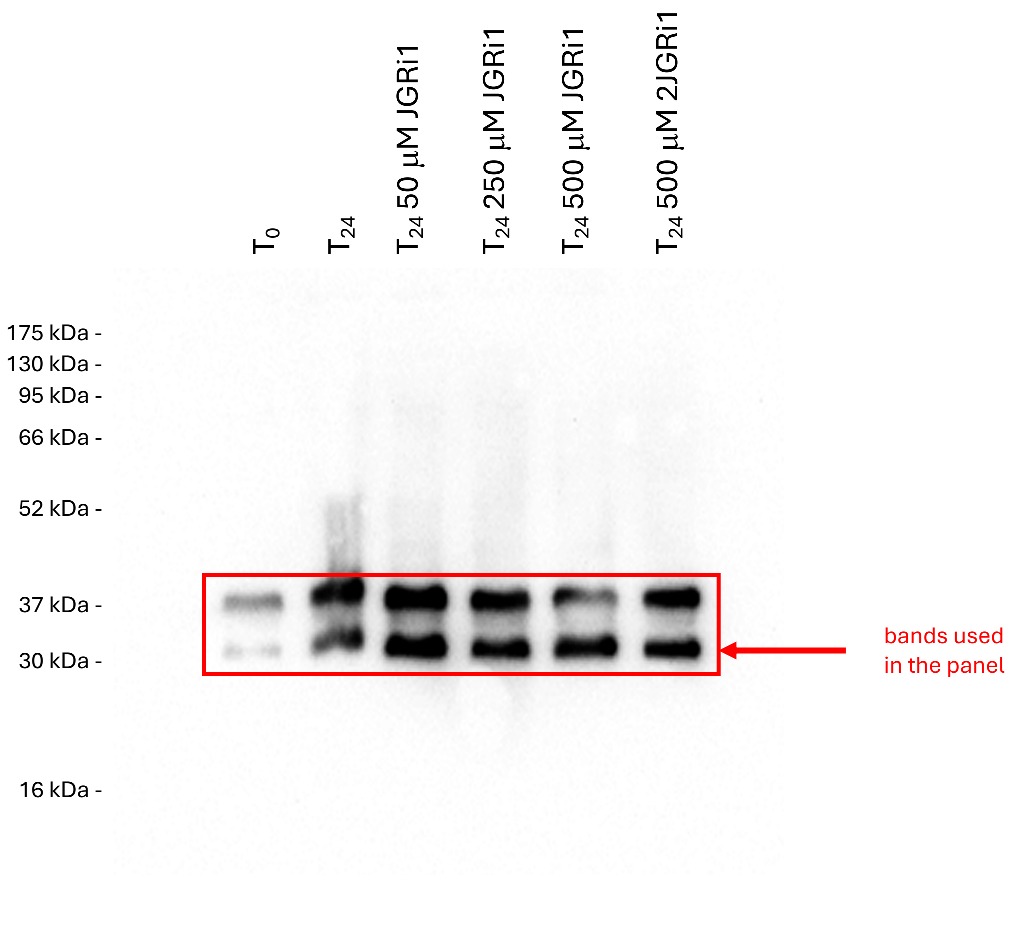

STX1A (35 kDa)

Fig.6D

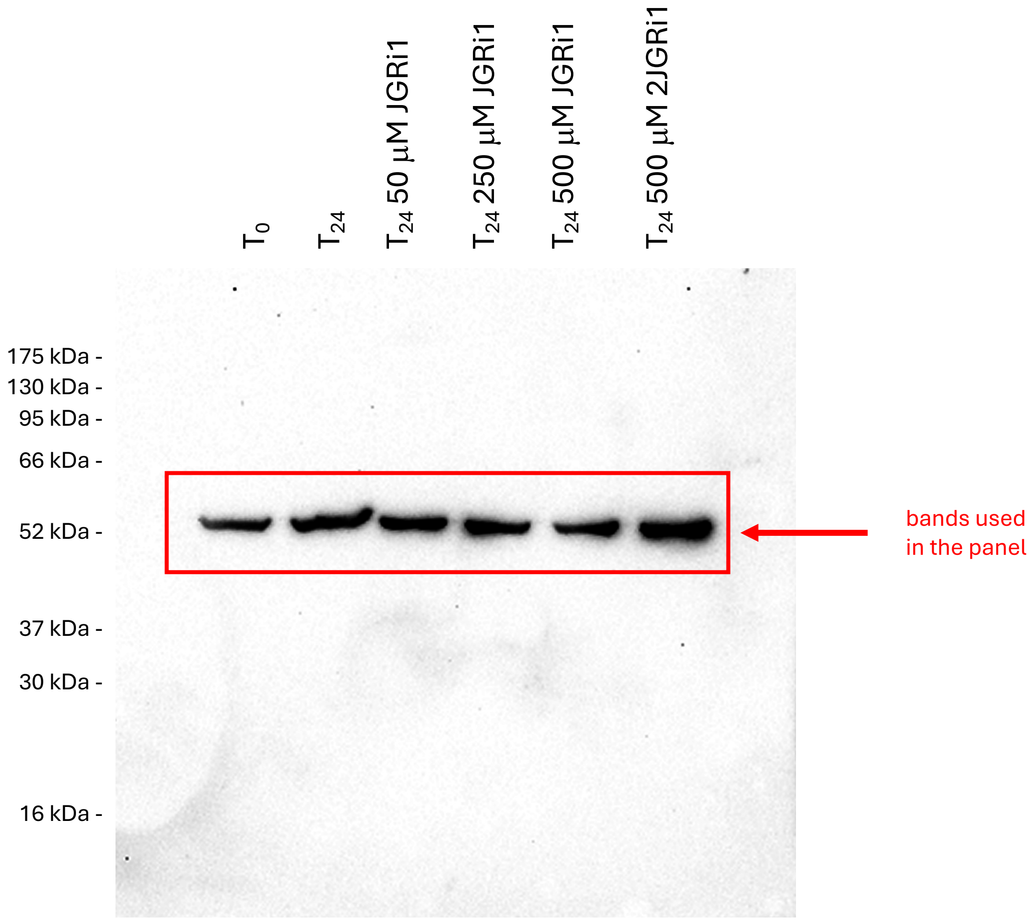

β-TUB (50 kDa)

Fig.6F

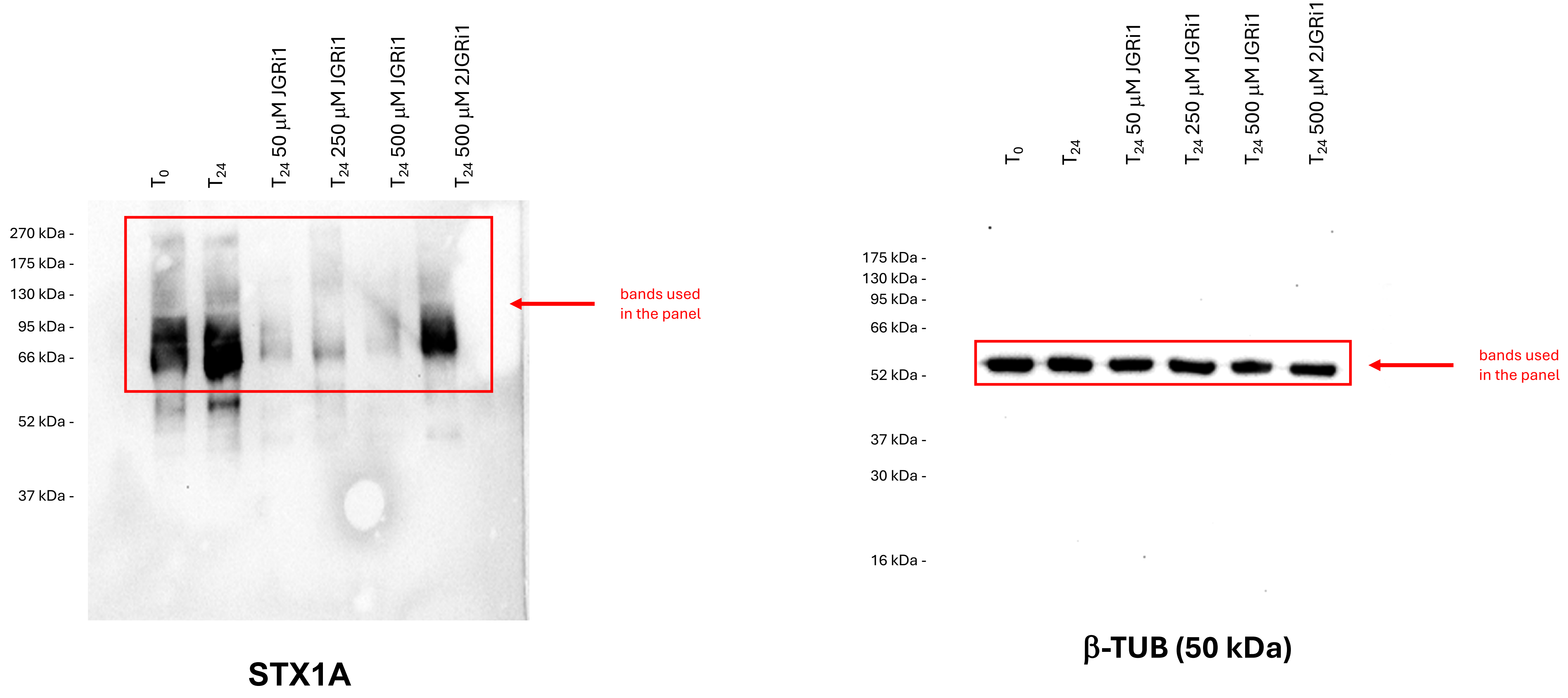
T0
T24
T24 50 μM JGRi1
T24 250 μM JGRi1
T24 500 μM JGRi1
T24 500 μM 2JGRi1
270 kDa -
175 kDa -
130 kDa -
95 kDa -
66 kDa -
52 kDa -
37 kDa -
bands used in the panel
STX1A
T0
T24
T24 50 μM JGRi1
T24 250 μM JGRi1
T24 500 μM JGRi1
T24 500 μM 2JGRi1
175 kDa -
130 kDa -
95 kDa -
66 kDa -
52 kDa -
37 kDa -
30 kDa -
16 kDa -
bands used in the panel
β-TUB (50 kDa)

Fig.S4B

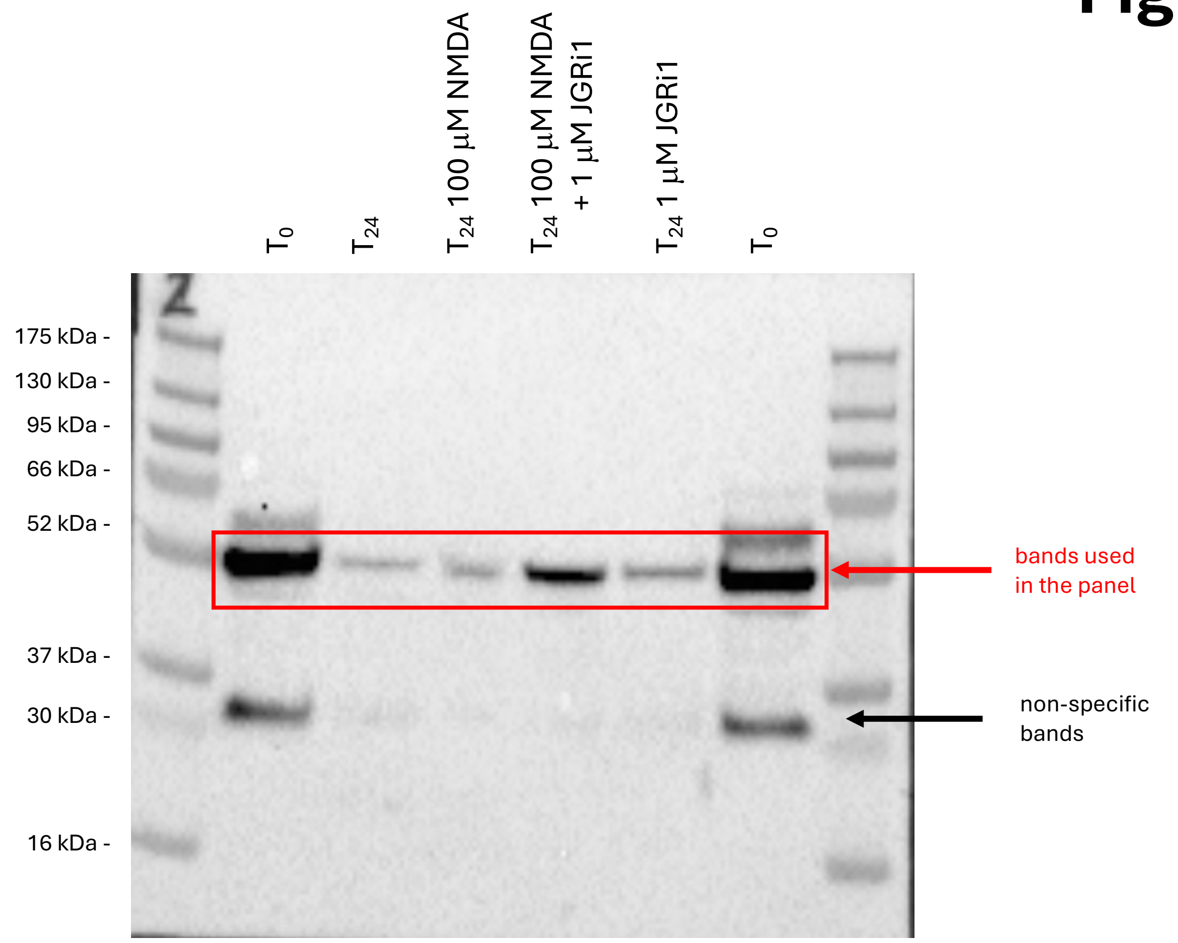

BRN3A (49 kDa)

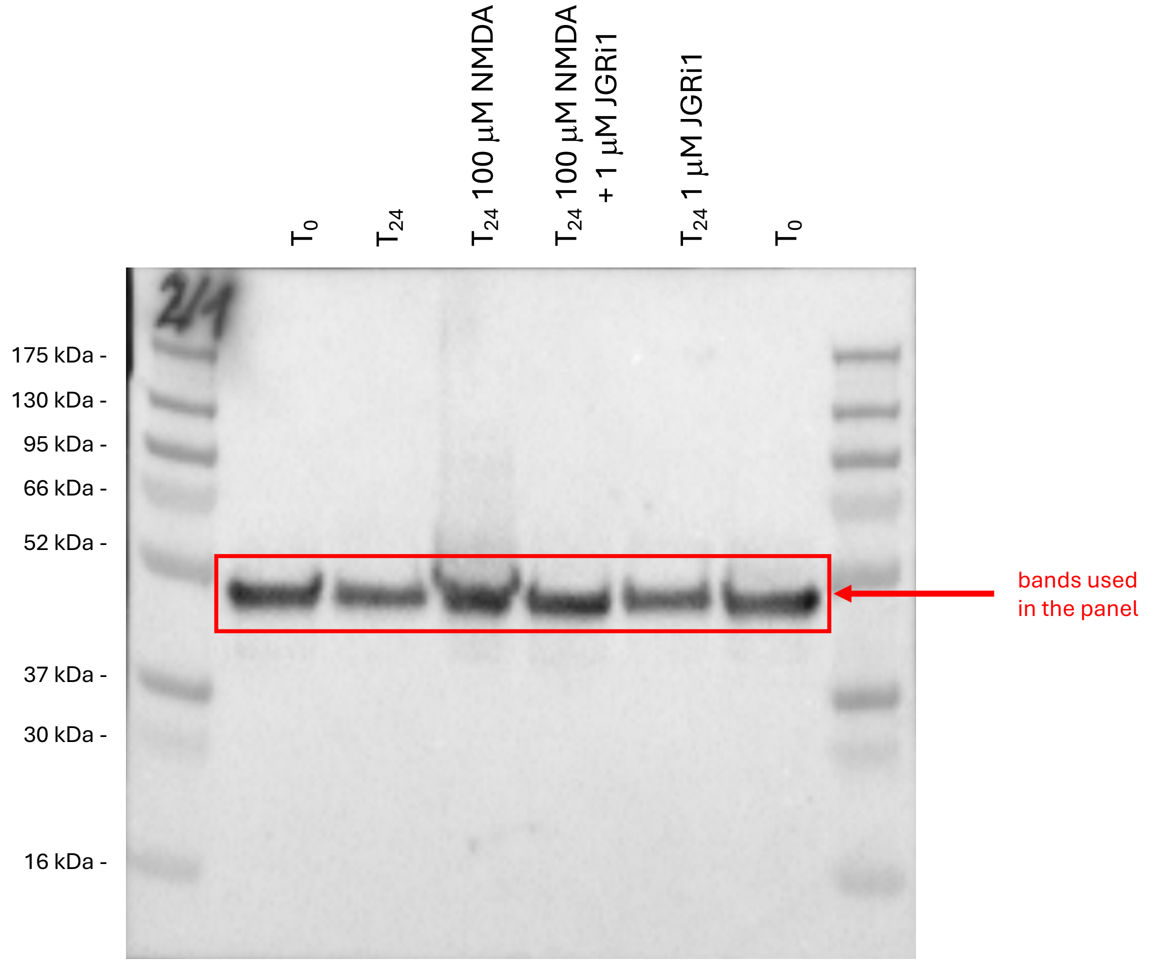

GS (44 kDa)

Fig.S4B

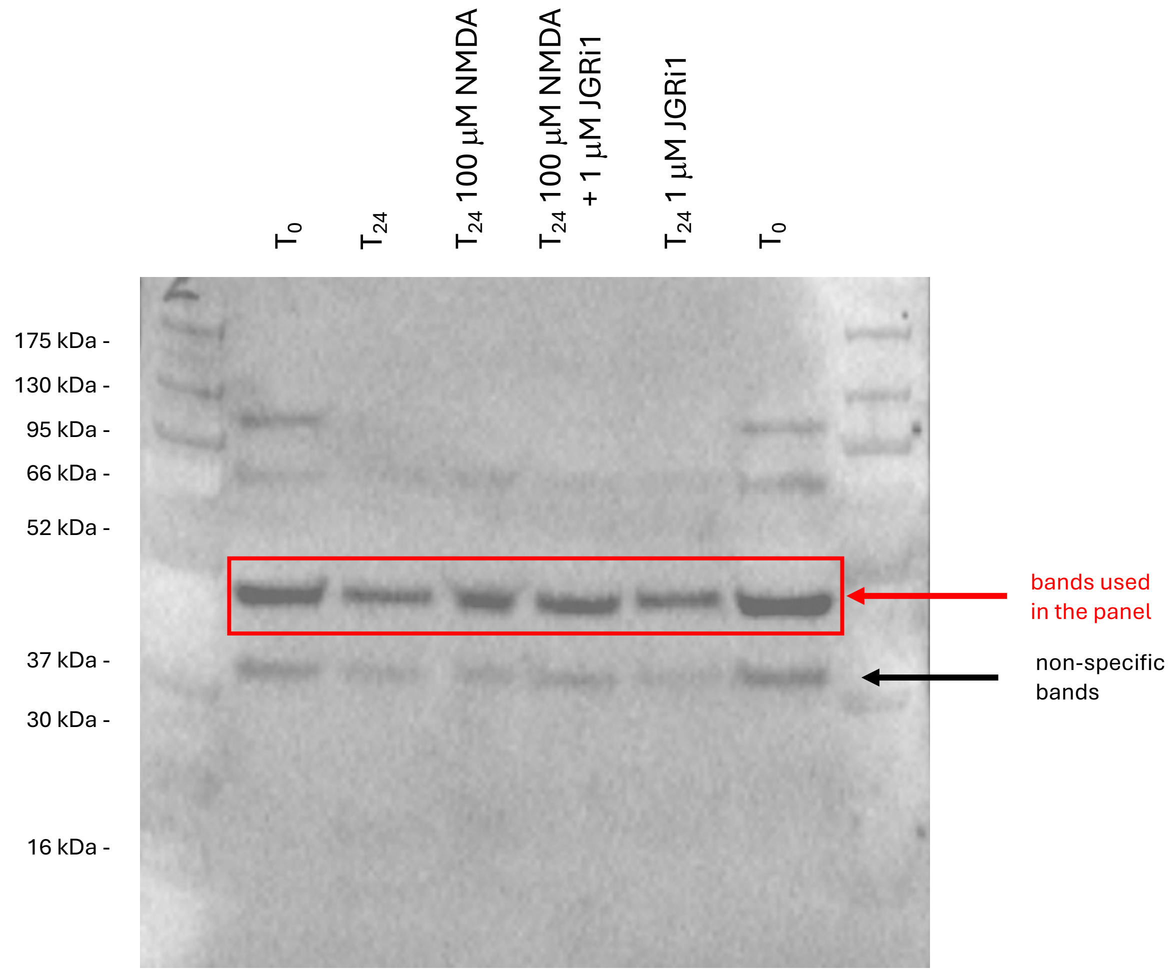

α-actin (42 kDa)
