# Supplementary material for: Topical eye treatment with JGRi1, a protein/protein interaction inhibitor, mitigates retinal degeneration
Source: Cell Death Dis. 2026 Apr 15;17(1):504. doi: 10.1038/s41419-026-08717-x (PMC13194685; doi:10.1038/s41419-026-08717-x)
Supplement: Supplementary file 2 — Supplementary figure legends [file 41419_2026_8717_MOESM2_ESM.docx]

**SUPPLEMENTARY FIGURE LEGENDS**

**Figure S1. Detection of retina autofluorescence and possible secondary antibody cross-reactivity. (A)** Representative images showing retinal autofluorescence upon exposure to different wavelengths (488 nm – 594 nm). White arrowheads in the merge column indicate areas where retinal autofluorescence localizes. n = 3 independent experiments. **(B)** Representative images showing potential secondary antibody cross-reactivity. Eyes were processed for immunofluorescence using only secondary antibodies, namely AlexFluor-488 (green) and AlexaFluor-594 (red). n = 3 independent experiments.

**Immunofluorescence caption:** OS, outer segment; ONL, outer nuclear layer; OPL, outer plexiform layer; INL, inner nuclear layer; IPL, inner plexiform layer; GCL, ganglion cell layer. 40X magnification. Scale bar 10 μM.

**Figure S2. NMDA treatment induces RGC degeneration and GS expression in retina wholemount preparations. (A)** Schematic representation of cultured retina wholemount preparations. **(B**) Representative picture of a cultured retinal wholemount preparation. **(C)** Representative western blot for BRN3A and GS. Samples were blotted, then incubated with a specific primary antibody against BRN3A, GS and β-TUB (loading control). n = 5 independent experiments. **(D)** Densitometric analysis of BRN3A with respect to β-TUB. **(E)** Densitometric analysis of GS with respect to β-TUB. Bar plots representing mean +/- S.D.

**Bar plot caption:** Bar representing mean +/- S.D; number of individual replicates per condition shown in the scatter plot. Statistical analysis: One-way ANOVA, two-tailed *post hoc* Tukey test, p < 0.05. p < 0.05. *p < 0.05; ** p < 0.01; *** p < 0.001; *** p < 0.0001.

**Figure S3. Fluorescently-labelled JGRi1 (F-JGRi1) penetrates the eye and accumulates in the retina of C57BL/6J mice upon *ex vivo* treatment. (A)** Schematic representation of F-JGRi1 treatment. **(B)** Representative immunofluorescence for F-JGRi1. n = 5 independent experiments. **(C)** Quantification of the mean fluorescence intensity of F-JGRi1 in the OPL. **(D)** Quantification of the mean fluorescence intensity of F-JGRi1 in the GCL. (**E)** Enlarged view of F-JGRi1 accumulation in the GCL. n = 5 independent experiments. **(F)** Quantification of F-JGRi1-positive cells.

**Immunofluorescence caption:** OS, outer segment; ONL, outer nuclear layer; OPL, outer plexiform layer; INL, inner nuclear layer; IPL, inner plexiform layer; GCL, ganglion cell layer. 40X magnification. Scale bar 10 μM.

**Bar plot caption:** Bar representing mean +/- S.D; number of individual replicates per condition shown in the scatter plot. Statistical analysis: One-way ANOVA, two-tailed *post hoc* Tukey test, p < 0.05. p < 0.05. *p < 0.05; ** p < 0.01; *** p < 0.001; *** p < 0.0001.

**Figure S4. In absence of retinal injury, topical JGRi1 does not affect the JNK2-STX1A interaction as well as glutamate levels in the retina of C57BL/6J mice. (A)** Schematic representation of in vivo JGRi1 treatment. **(B)** Representative co-immunofluorescence for JNK2 and STX1A. Eyes were processed for immunofluorescence with a JNK2-specific antibody (green) and a STX1A-specific antibody (red). n = 3 independent experiments. **(C)** Quantification of the mean fluorescence intensity of JNK2. **(D)** Quantification of the mean fluorescence intensity of STX1A. **(E)** Correlation analysis between JNK2 and STX1A signals. Pearson’s scores were calculated per each image from (B). **(F)** Representative immunofluorescence for L-glut. Eyes were processed for immunofluorescence with an L-glut-specific antibody (green). n = 3 independent experiments. **(G)** Quantification of the mean fluorescence intensity of L-glut.

**Immunofluorescence caption:** OS, outer segment; ONL, outer nuclear layer; OPL, outer plexiform layer; INL, inner nuclear layer; IPL, inner plexiform layer; GCL, ganglion cell layer. 40X magnification. Scale bar 10 μM.

**Bar plot caption:** Bar representing mean +/- S.D; number of individual replicates per condition shown in the scatter plot. Statistical analysis: One-way ANOVA, two-tailed *post hoc* Tukey test, p < 0.05. p < 0.05. *p < 0.05; ** p < 0.01; *** p < 0.001; *** p < 0.0001.

**Figure S5. Topical JGRi1 preserves PROX1-positive cells in INL and prevents glutamate accumulation at synaptic level in evONC model. (A)** Representative immunofluorescence for PROX1. Eyes were processed for immunofluorescence with an PROX1-specific antibody (green). n = 3 independent experiments. **(B)** Quantification of PROX1-positive cells. **(C)** Representative co-immunofluorescence for SYT1 and L-glut. Eyes were processed for immunofluorescence with a SYT1-specific antibody (green) and a L-glut-specific antibody (red). n = 3 independent experiments. **(D)** Correlation analysis between SYT1 and L-glut signals. Pearson’s scores were calculated per each image from (C).

**Immunofluorescence caption:** OS, outer segment; ONL, outer nuclear layer; OPL, outer plexiform layer; INL, inner nuclear layer; IPL, inner plexiform layer; GCL, ganglion cell layer. 40X magnification. Scale bar 10 μM.

**Bar plot caption:** Bar representing mean +/- S.D; number of individual replicates per condition shown in the scatter plot. Statistical analysis: One-way ANOVA, two-tailed *post hoc* Tukey test, p < 0.05. p < 0.05. *p < 0.05; ** p < 0.01; *** p < 0.001; *** p < 0.0001.

**Figure S6. JGRi1 prevents NMDA-induced loss of RGCs and boost in GS expression in retina wholemounts. (A)** Schematic representation of JGRi1 treatment on retina whole-mount preparations. **(B)** Representative western blot showing the expression of BRN3A and GS in wholemount retinas. Samples were blotted, then incubated with primary antibodies against BRN3A, GS and, ultimately, α-actin (loading control). n = 5 independent experiments. **(C)** Densitometric analysis of BRN3A with respect to α-actin. **(D)** Densitometric analysis of glutamine synthetase with respect to α-actin.

**Bar plot caption:** Bar representing mean +/- S.D; number of individual replicates per condition shown in the scatter plot. Statistical analysis: One-way ANOVA, two-tailed *post hoc* Tukey test, p < 0.05. p < 0.05. *p < 0.05; ** p < 0.01; *** p < 0.001; *** p < 0.0001.

**Figure S7. Topical JGRi1 preserves PROX1-positive cells in INL and prevents glutamate accumulation at synaptic level upon NMDA injection. (A)** Representative immunofluorescence for PROX1. Eyes were processed for immunofluorescence with an PROX1-specific antibody (green). n = 3 independent experiments. **(B)** Quantification of PROX1-positive cells. **(C)** Representative co-immunofluorescence for SYT1 and L-glut. Eyes were processed for immunofluorescence with a SYT1-specific antibody (green) and a L-glut-specific antibody (red). n = 3 independent experiments. **(D)** Correlation analysis between SYT1 and L-glut signals. Pearson’s scores were calculated per each image from (C).

**Immunofluorescence caption:** OS, outer segment; ONL, outer nuclear layer; OPL, outer plexiform layer; INL, inner nuclear layer; IPL, inner plexiform layer; GCL, ganglion cell layer. 40X magnification. Scale bar 10 μM.

**Bar plot caption:** Bar representing mean +/- S.D; number of individual replicates per condition shown in the scatter plot. Statistical analysis: One-way ANOVA, two-tailed *post hoc* Tukey test, p < 0.05. p < 0.05. *p < 0.05; ** p < 0.01; *** p < 0.001; *** p < 0.0001.
