# Supplementary figures and images for: Topical eye treatment with JGRi1, a protein/protein interaction inhibitor, mitigates retinal degeneration
Source: Cell Death Dis. 2026 Apr 15;17(1):504. doi: 10.1038/s41419-026-08717-x (PMC13194685; doi:10.1038/s41419-026-08717-x)

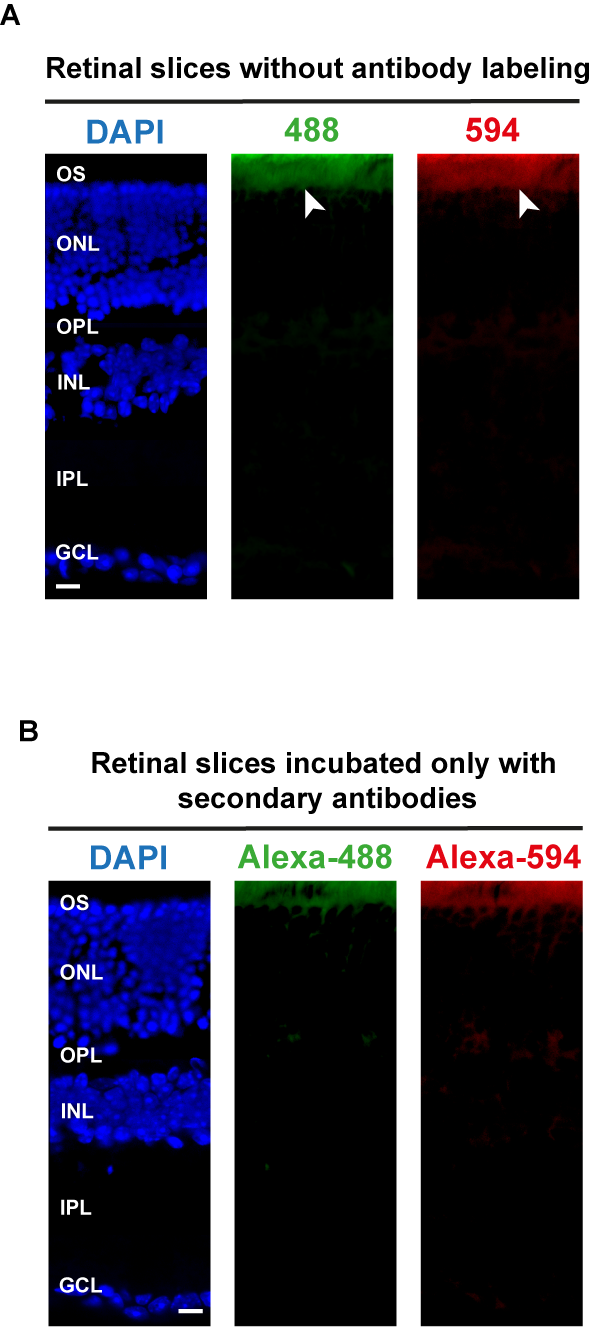

Supplement: Supplementary file 3 — Figure S1 [file 41419_2026_8717_MOESM3_ESM.tif]

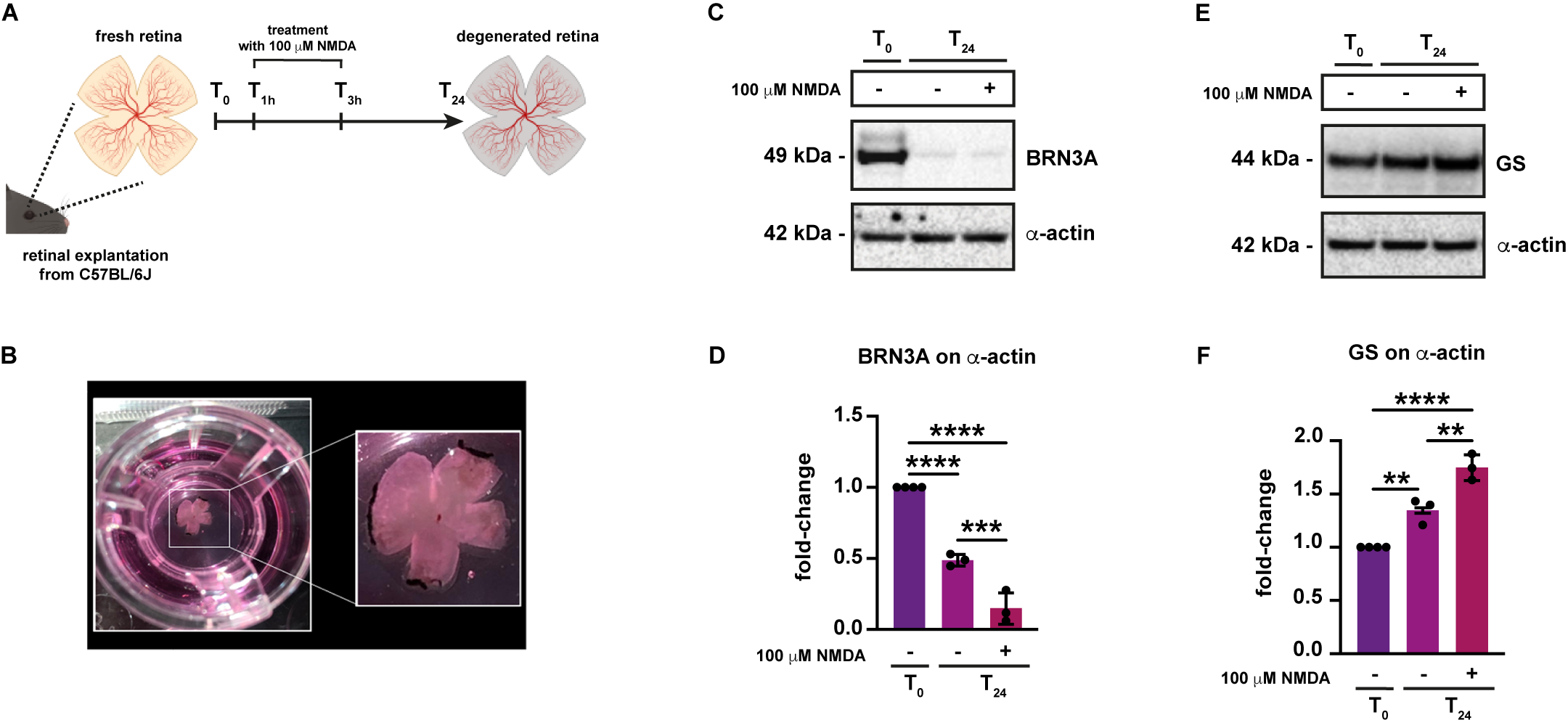

Supplement: Supplementary file 4 — Figure S2 [file 41419_2026_8717_MOESM4_ESM.tif]

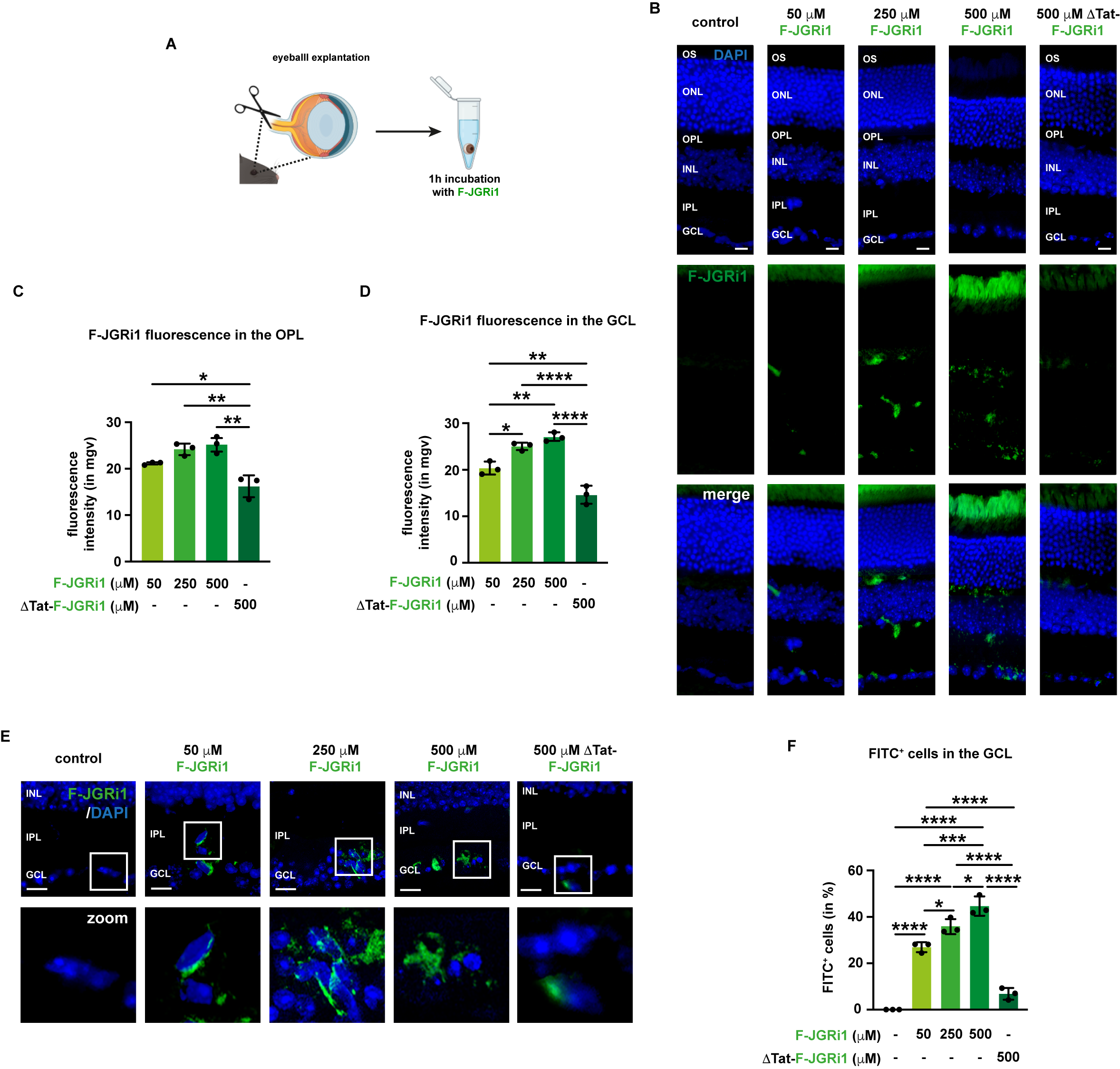

Supplement: Supplementary file 5 — Figure S3 [file 41419_2026_8717_MOESM5_ESM.tif]

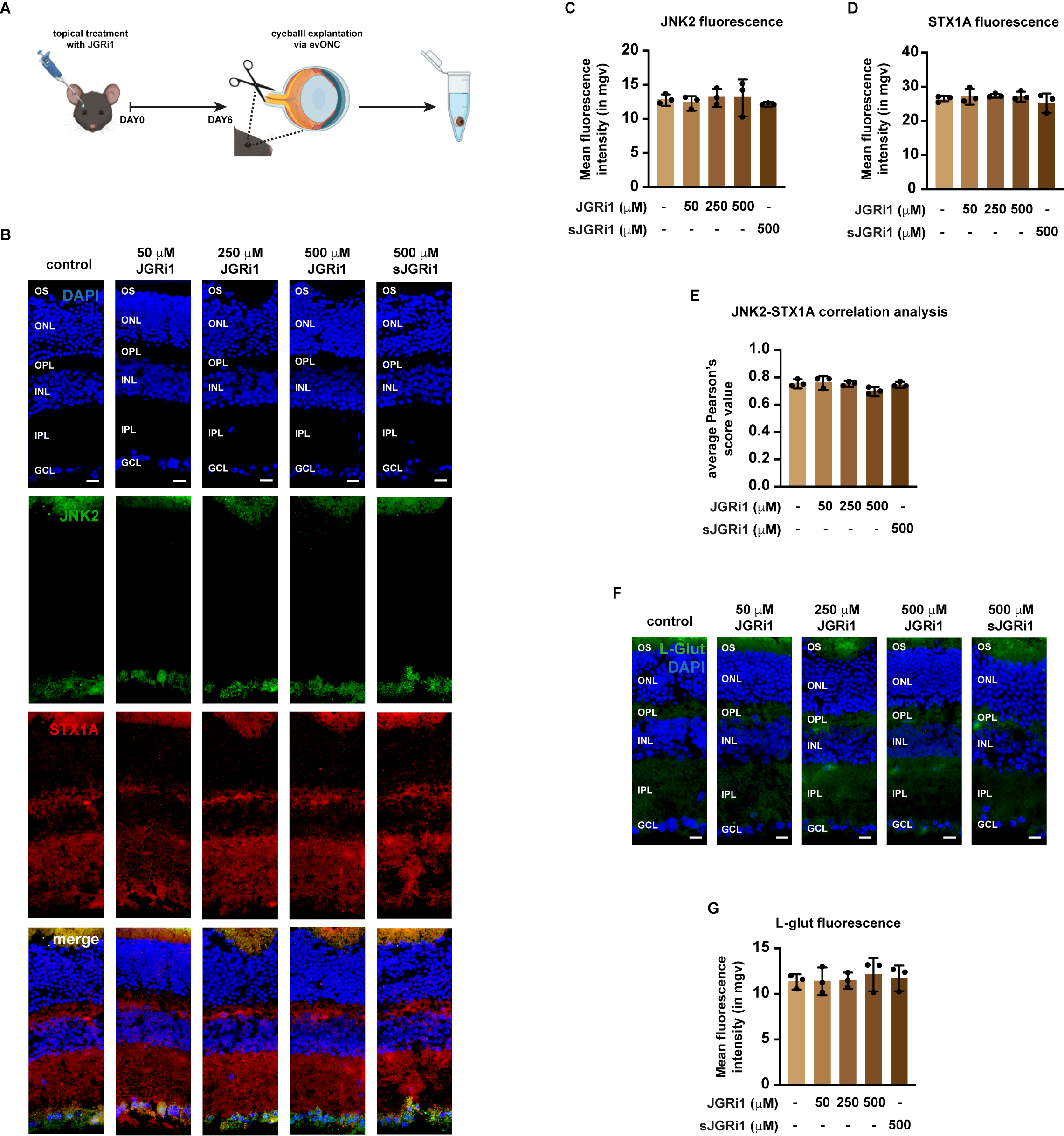

Supplement: Supplementary file 6 — Figure S4 [file 41419_2026_8717_MOESM6_ESM.tif]

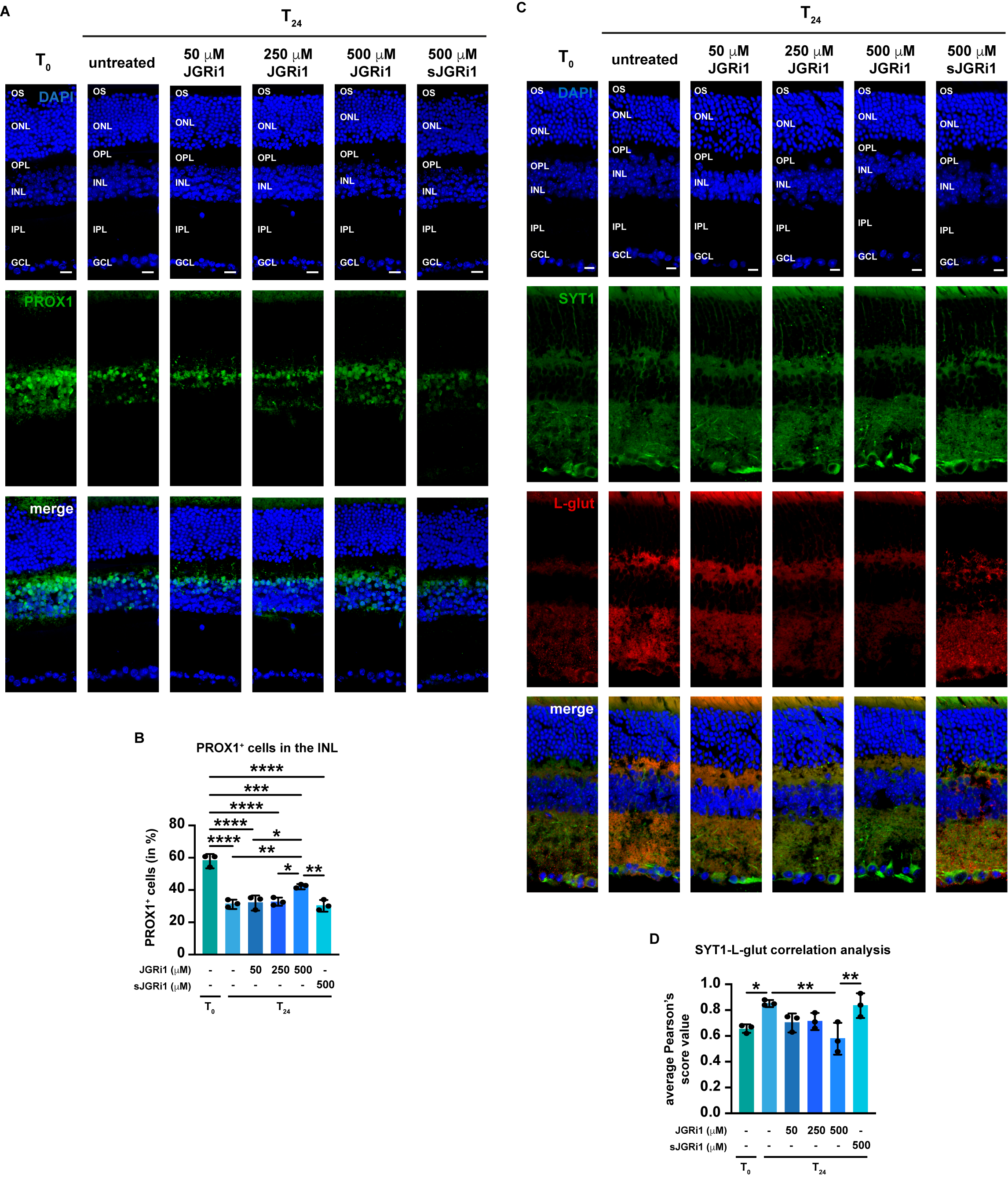

Supplement: Supplementary file 7 — Figure S5 [file 41419_2026_8717_MOESM7_ESM.tif]

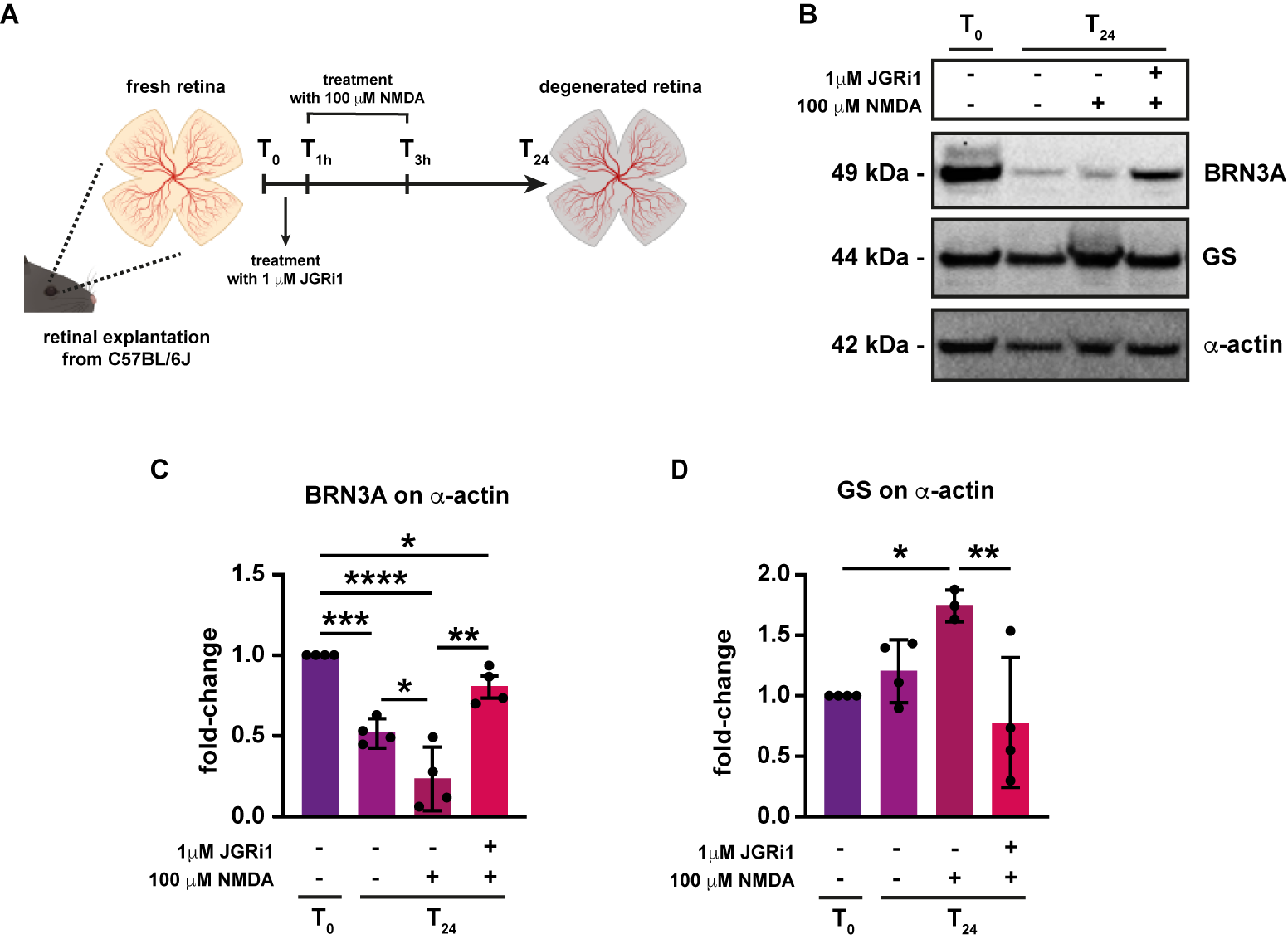

Supplement: Supplementary file 8 — Figure S6 [file 41419_2026_8717_MOESM8_ESM.tif]

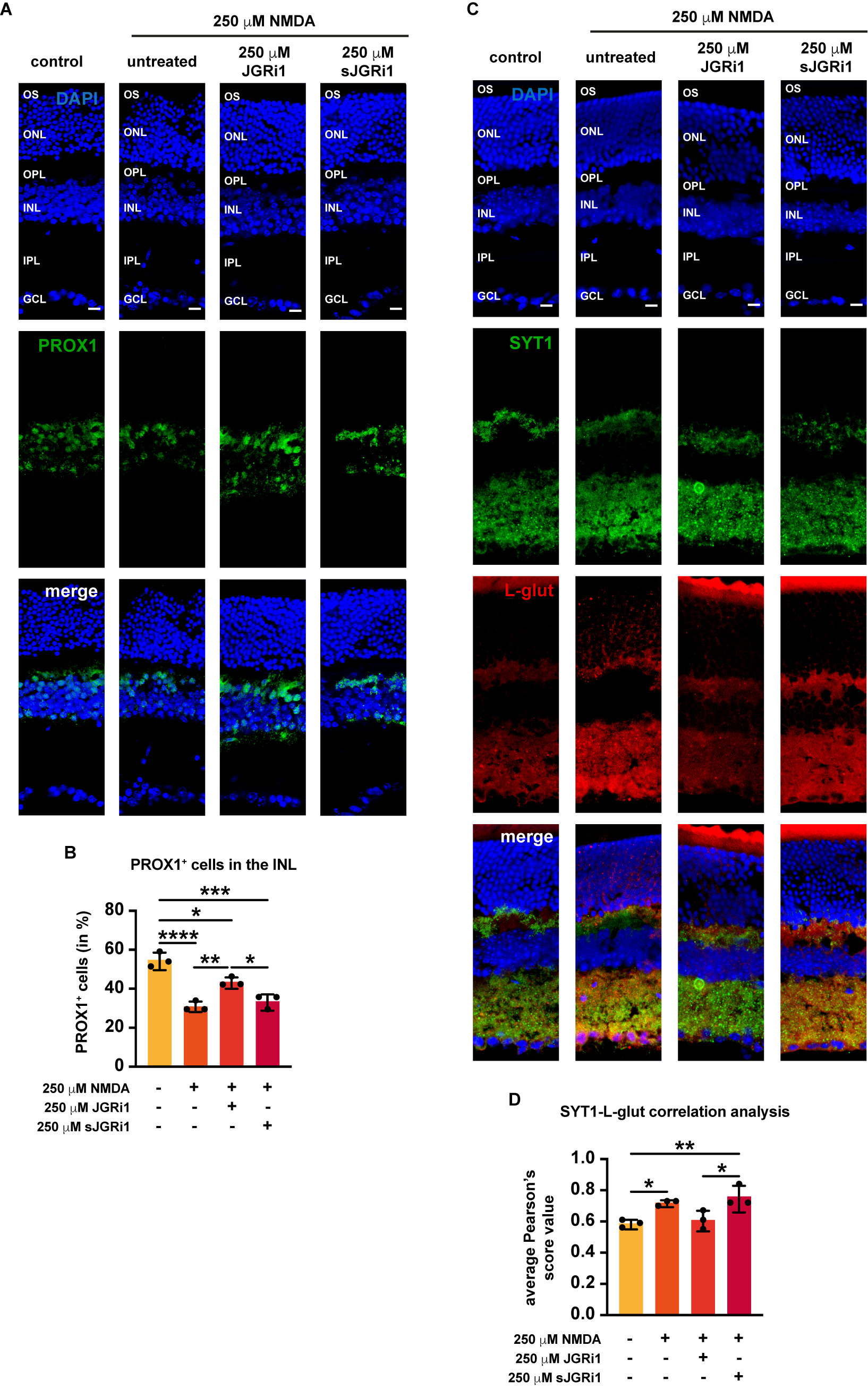

Supplement: Supplementary file 9 — Figure S7 [file 41419_2026_8717_MOESM9_ESM.tif]
